# Metabolic Profiling of Individuals with Missing Teeth and Tooth Loss

**DOI:** 10.1177/00220345241298219

**Published:** 2025-01-03

**Authors:** A.M. Halme, A. Salminen, A.L. Suominen, A. Havulinna, P. Mäntylä, K. Buhlin, S. Paju, S. Männistö, V. Salomaa, W. Sattler, J. Sinisalo, P.J. Pussinen

**Affiliations:** 1 Oral and Maxillofacial Diseases, University of Helsinki and Helsinki University Hospital, Helsinki, Finland; 2 Institute of Dentistry, University of Eastern Finland, Kuopio, Finland; 3 Oral and Maxillofacial Teaching Unit, Kuopio University Hospital, Kuopio, Finland; 4 National Institute for Health and Welfare, Helsinki, Finland; 5 Institute for Molecular Medicine Finland, Helsinki Institute of Life Science, University of Helsinki, Helsinki, Finland; 6 Department of Dental Medicine, Karolinska Institutet, Huddinge, Sweden; 7 Division of Molecular Biology and Biochemistry, Gottfried Schatz Research Center, Medical University of Graz, Graz, Austria; 8 HUCH Heart and Lung Center, Helsinki University Hospital and University of Helsinki, Helsinki, Finland

**Keywords:** metabolomics, lipoprotein, fatty acid, oral health, cardiovascular disease, dyslipidemias

## Abstract

Missing teeth have been linked to incident cardiovascular disease, diabetes, and all-cause mortality. Our previous study revealed that signs of oral infections and inflammatory conditions (i.e., periodontal disease and dental caries) are associated with disadvantageous features of circulating metabolites. This study investigates whether missing teeth and tooth loss, the end points of these diseases, are associated with similar metabolic features. The 2 Finnish population-based studies Health-2000 (*n* = 6,197) and FINRISK-97 (*n* = 6,050) were included, as was Parogene (*n* = 465), a cohort of patients with an indication for coronary angiography. The number of teeth was recorded in clinical examinations. Serum concentrations of 157 metabolites were determined by a nuclear magnetic resonance spectroscopy–based method. Health-2000 participants (*n* = 3,371) provided follow-up serum samples, and 1,186 of them participated in a repeated oral examination 11 y after the baseline. Linear regression models adjusted for age, sex, smoking, body mass index, and diabetes were fitted to the number of teeth and metabolite measures. The results from the separate cohorts were combined in a fixed-effects meta-analysis. We also analyzed whether the number of teeth at baseline and tooth loss during follow-up were associated with changes in metabolite concentrations. Missing teeth were associated with increased very-low-density lipoprotein–related measures and triglyceride concentrations, as well as with decreased high-density lipoprotein parameters and small particle size. Missing teeth also had an association with low levels of unsaturated fatty acids (FAs), including omega-3 and omega-6 FAs, and elevated proportions of monounsaturated and saturated FAs. The number of teeth at baseline predicted changes in several concentrations, such as measures related to intermediate-density lipoprotein, low-density lipoprotein, and FAs, but no associations with tooth loss during the 11-y follow-up were observed. To conclude, missing teeth are associated with adverse metabolic features characterized by systemic inflammation and several risk factors for cardiometabolic diseases.

## Introduction

Metabolomics of different biofluids provides measures of small molecules, such as substrates and products of metabolism, deriving from the host and microbes. The metabolic fingerprints represent molecular phenotypes related to physiologic and pathologic processes. Ideally, and especially when combined with other omics techniques, metabolomics may lead to the identification of new biomarkers for specific health conditions or the revelation of pathogenic mechanisms (Babu and Snyder 2023).

Metabolomics of oral samples, including gingival crevicular fluid or saliva, in relation to missing teeth or tooth loss has been investigated in few studies (Liebsch et al. 2019; Andörfer et al. 2021). Saliva metabolites originating from tissue destruction measured by mass spectrometry were associated with periodontal parameters in young and middle-aged groups, whereas tooth loss showed a strong association with the metabolite concentrations in older participants (≥60 y; Liebsch et al. 2019). Among 84 metabolites linked to oral health parameters, the concentrations of 14 (16.7%) were associated with missing teeth, and 9 (26.5%) of 34 candidate metabolites that were associated with at least 2 oral parameters presented an association with 5-y tooth loss (Andörfer et al. 2021).

While saliva metabolites largely reflect local tissue destruction and metabolism of oral microbiota, serum metabolomics describes systemic health more comprehensively. However, a limited number of studies have examined relationships between missing teeth and circulating metabolites. Mewes et al. (2022) found an association between serum docosapentaenoic acid and tooth loss in patients with periodontitis. Another study suggested a link between periodontal health and fish consumption (Ottosson et al. 2022).

The nuclear magnetic resonance (NMR) platform provides >150 metabolic measures, including concentrations and compositions of lipoprotein particles, apolipoproteins, fatty acids (FAs), amino acids, glycolysis-derived molecules, and ketone bodies. Several of these metabolites are associated with immunoinflammatory responses and thus increased risk of cardiovascular events or neurodegenerative diseases (Wang et al. 2011; Würtz et al. 2015; Nath et al. 2017; Quintero Escobar et al. 2021).

We recently demonstrated that signs of oral infections are associated with disadvantageous and inflammatory metabolic features, with alterations especially in metabolites related to very-low-density lipoprotein (VLDL) and high-density lipoprotein (HDL) (Salminen et al. 2024). Notably, caries- and periodontitis-related parameters displayed significant associations in cross-sectional and prospective analyses. Thus, the 2 main causes of tooth loss accompany adverse systemic metabolic signatures. Tooth loss is considered an end point of both diseases.

Extraction of nonvital teeth may support achieving optimal C-reactive protein levels (Miller et al. 2023). However, missing teeth are associated with incident coronary artery disease events, acute myocardial infarction, cardiovascular disease (CVD), diabetes, and all-cause mortality (Liljestrand et al. 2015). Missing teeth were also associated with multiple CVD risk factors, including C-reactive protein, low HDL cholesterol, and triglycerides (Liljestrand et al. 2015). These results suggest that losing teeth (i.e., removing the cause of local inflammation) promotes no improvement in CVD risk. Therefore, the present study analyzes the systemic metabolic features obtained by NMR spectroscopy in relation to missing teeth and alterations in metabolite concentrations predicted by tooth loss.

## Materials and Methods

### Parogene

The original COROGENE study included 5,297 patients who underwent coronary angiography for any indication in Helsinki University Hospital, Helsinki, Finland, between 2006 and 2008 (Vaara et al. 2012). The ethics committee of Helsinki University Hospital (HUS/152/2016) approved the research plan, and all study participants signed an informed consent form. A random sample of 10% of patients agreed to participate in the Parogene substudy (Buhlin et al. 2011). In Parogene, 506 participants attended an extensive clinical oral examination that was performed by a dentist and included blood sampling.

### Health-2000 and Health-2011

The nationwide Health-2000 survey is based on a stratified 2-stage cluster sample of 8,028 ≥30-y-old Finns. Data collection involved structured health interviews, blood sampling, and clinical oral examinations, which were performed on 6,335 participants by dentists (Suominen-Taipale et al. 2008).

Health-2011 is a follow-up survey based on Health-2000 (Joshi et al. 2018). All participants of the original study who were alive and residing in Finland were invited to attend. Only adults living in Southern or Northern Finland were invited to a repeated clinical oral examination, and 1,496 (41%) agreed to participate. The follow-up length was 11 y (mean, 10.8 y; range, 10.2 to 11.2 y). The ethics committees of Helsinki University Hospital and the National Public Health Institute approved the study protocols, and each participant signed an informed consent form.

### FINRISK-97

The National FINRISK 1997 Study (FINRISK-97) is a national population-based cohort (n = 8,446) with participants aged 25 to 74 y (Vartiainen et al. 2010). The survey methods follow the World Health Organization’s MONICA protocol (WHO MONICA Project Principal Investigators 1988). The participants filled in a comprehensive questionnaire and participated in a clinical examination, in which a trained nurse counted the number of teeth using a flashlight and a spatula. The ethics committee of the National Public Health Institute approved the study protocol, and all participants gave informed consent.

### NMR Metabolomics

Nightingale Health (Helsinki, Finland) analyzed the serum samples of all cohorts with the high-throughput NMR metabolomics platform described previously (Würtz et al. 2017). The protocol gave data on 157 metabolites, including concentrations and compositions of lipoprotein particles divided into subclasses by density and diameter; concentrations and proportions of FAs; and measures of amino acids, ketone bodies, and other molecules involved in cell metabolism (Soininen et al. 2015). NMR measurements were available for 465 participants in Parogene, 6,229 in Health-2000, 3,371 in the Health-2011 follow-up study (1,186 of whom had their number of teeth recorded), and 6,050 in FINRISK-97.

### Statistical Analyses

Statistical analyses were performed with R (version 3.6.1 or higher; http://www.r-project.org/). The threshold for statistical significance was set at 0.05/157 = 0.00032 to correct for multiple testing.

To obtain approximately normal distributions, the metabolite concentrations were log(*x*+1) transformed and scaled to a mean of 0 and an SD of 1. We fitted linear regression models with metabolic concentrations as outcomes and the number of teeth as a predictor separately in all cohorts. Oral disease risk factors that could be harmonized among cohorts were applied as covariates. These included age, sex, smoking status (current, former, never smoker), body mass index, and information on type 1 or 2 diabetes mellitus, as gathered from registers and questionnaires. An inverse variance–weighted fixed-effect meta-analysis (R package *meta*) combined the results from the 3 distinct cohorts.

In Health-2000/Health-2011, linear regression models were fitted between the follow-up metabolite concentrations and the number of teeth at baseline or teeth lost during the 11-y follow-up. The analyses were adjusted for baseline metabolite concentrations and the latter analysis also for the original tooth count. The baseline number of teeth was used as a continuous variable in cross-sectional and prospective analyses, whereas tooth loss was converted to a binomial form with the alternatives “no teeth lost” and “≥1 tooth lost.” We defined complete dentition as 32 teeth and missing teeth as an absence of 1 or more teeth for any reason. The study was conducted following the STROBE guidelines (Strengthening the Reporting of Observational Studies in Epidemiology).

## Results

Altogether 12,712 participants from 3 distinct cohorts were employed in the cross-sectional part of the study (Table 1). In FINRISK-97 and Health-2000, approximately half of the participants were women (49.1% and 54.9%, respectively), whereas Parogene comprised 35.1% women. The mean age in Parogene was higher than in FINRISK-97 and Health-2000. All cohorts had a substantially aligned median tooth count, yet the proportion of individuals who were edentulous was considerably lower in Parogene than in other populations.

**Table 1.** Characteristics of Study Populations.

	Cross-sectional			Follow-up (*n* = 1,186)	
	Parogene (*n* = 465)	FINRISK-97 (*n* = 6,050)	Health-2000 (*n* = 6,197)	Health-2000	Health-2011
Mean (SD)					
Age, y	63.6 (9.0)	53.2 (10.6)	52.8 (14.9)	48.0 (11.3)	59.0 (11.3)
Body mass index, kg/m^2^	27.8 (5.0)	27.2 (4.5)	26.9 (4.6)	26.3 (4.4)	27.1 (4.7)
Lost teeth					0.96 (2.1)
Median (IQR)					
No. of teeth	23 (15 to 27)	25 (7 to 30)	25 (9 to 28)	27 (21 to 28)	26 (19 to 28)
Participants, *n* (%)					
Women	163 (35.1)	2,971 (49.1)	3,383 (54.9)	660 (55.6)	660 (55.6)
Smoking					
Current	55 (11.8)	1,362 (22.5)	1,629 (26.3)	309 (26.1)	209 (17.6)
Former	187 (40.2)	1,507 (24.9)	1,350 (21.8)	259 (21.8)	339 (28.6)
Never	222 (47.7)	3,181 (52.6)	3,192 (51.5)	613 (51.2)	625 (52.7)
Diabetes	108 (23.2)	434 (7.2)	340 (5.5)	34 (2.9)	87 (7.3)
Edentulous	29 (6.2)	977 (16.1)	917 (14.8)	83 (7.0)	98 (8.3)

In the Health-2000 survey, 3,371 participants attended the follow-up and gave blood samples for metabolite profiling 11 y after baseline. Half of them (*n* = 1,186) underwent a repeated oral examination. Altogether 458 participants had lost at least 1 tooth, and 1,144 teeth were extracted over the 11-y period. The mean number of remaining teeth was 22.6 at baseline and 21.7 at the follow-up. Most commonly, only 1 tooth (*n* = 219) or 2 teeth (*n* = 108) were lost.

**Figure 1.**
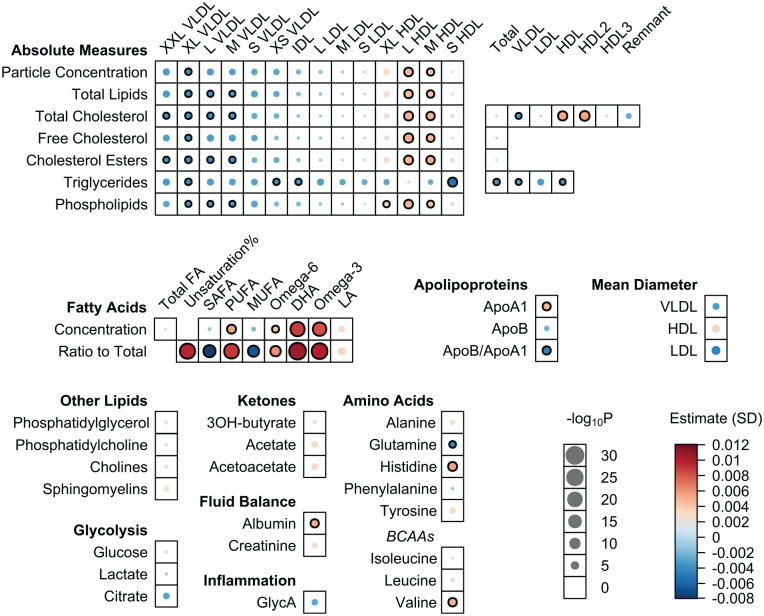
Associations between number of teeth and metabolites acquired from the meta-analysis. From Parogene, FINRISK-97, and Health-2000 cohorts, concentrations of 157 metabolites were determined by a nuclear magnetic resonance platform. The associations were first investigated separately in each cohort, and the results were then combined by a fixed-effects meta-analysis. The analyses were adjusted for age, sex, smoking status (never, former, current), body mass index, and diabetes. Significant findings (*P* < 0.00032) are indicated with black circles. ApoA1, apolipoprotein A-I; ApoB, apolipoprotein B; BCAA, branched-chain amino acid; DHA, docosahexaenoic acid; FA, fatty acid; GlycA, glycoprotein acetyls; HDL, high-density lipoprotein; IDL, intermediate-density lipoprotein; LA, linoleic acid; LDL, low-density lipoprotein; MUFA, monounsaturated fatty acid; PUFA, polyunsaturated fatty acid; SAFA, saturated fatty acid; VLDL, very-low-density lipoprotein.

The associations of 157 metabolic concentrations with the number of teeth were analyzed with a linear regression model (Appendix Table 1), and the results from the 3 cohorts were combined by a fixed-effect meta-analysis (Table 2, Appendix Table 2). Figure 1 illustrates these results. The number of teeth was significantly associated with 60 metabolites (38%, *P* < 0.00032; Table 2). The results from the distinct cohorts were mainly aligned, and estimates displayed the same direction (beta values from individual cohorts are presented in Appendix Table 1).

**Table 2.** Significant Associations between Number of Teeth and Metabolites in the Meta-analysis of 3 Cohorts.

		Meta-analysis			Heterogeneity	
Target: Subclass	Metabolite	β	SD	*P* Value	*I* ^2^	*P* Value
**VLDL**						
XXL	Cholesterol	−0.0034	0.00090	0.00019	0.63	0.065
	CE	−0.0034	0.00090	0.00013	0.61	0.075
XL	Particles	−0.0036	0.00090	8.2∙10^−5^	0.71	0.032
	Total lipids	−0.0036	0.00090	5.2∙10^−5^	0.67	0.047
	Cholesterol	−0.0036	0.00090	5.5∙10^−5^	0.66	0.054
	FC	−0.0034	0.00090	0.00015	0.62	0.070
	CE	−0.0037	0.00090	4.1∙10^−5^	0.70	0.034
	Triglycerides	−0.0036	0.00090	6.2∙10^−5^	0.71	0.032
	Phospholipids	−0.0034	0.00090	0.00019	0.62	0.073
L	Total lipids	−0.0033	0.00088	0.00016	0.59	0.085
	Cholesterol	−0.0035	0.00089	9.5∙10^−5^	0.67	0.047
	CE	−0.0035	0.00089	7.6∙10^−5^	0.68	0.043
	Phospholipids	−0.0032	0.00089	0.00028	0.65	0.055
M	Total lipids	−0.0032	0.00087	0.00023	0.42	0.18
	Cholesterol	−0.0034	0.00088	0.00012	0.47	0.15
	CE	−0.0035	0.00088	8.4∙10^−5^	0.46	0.16
	Phospholipids	−0.0032	0.00087	0.00023	0.51	0.13
XS	Triglycerides	−0.0034	0.00088	0.00011	0.00	0.60
**IDL**	Triglycerides	−0.0035	0.00089	0.00011	0.00	0.82
**HDL**						
XL	Phospholipids	0.0033	0.00084	0.00010	0.00	0.53
L	Particles	0.0047	0.00085	4.8∙10^−8^	0.00	0.55
	Total lipids	0.0045	0.00085	1.1∙10^−7^	0.00	0.56
	Cholesterol	0.0048	0.00084	1.5∙10^−8^	0.00	0.51
	FC	0.0050	0.00084	3.6∙10^−9^	0.00	0.39
	CE	0.0048	0.00084	1.9∙10^−8^	0.00	0.54
	Phospholipids	0.0047	0.00085	4.8∙10^−8^	0.00	0.49
M	Particles	0.0043	0.00095	6.4∙10^−6^	0.00	0.46
	Total lipids	0.0044	0.00095	3.6∙10^−6^	0.00	0.46
	Cholesterol	0.0049	0.00093	1.6∙10^−7^	0.00	0.48
	FC	0.0046	0.00094	1.3∙10^−6^	0.22	0.28
	CE	0.0049	0.00093	1.2∙10^−7^	0.00	0.54
	Phospholipids	0.0040	0.00095	2.1∙10^−5^	0.00	0.46
	Triglycerides	−0.0051	0.00089	1.1∙10^−8^	0.00	0.52
**Cholesterol**	VLDL	−0.0032	0.00086	0.00026	0.048	0.35
	HDL	0.0051	0.00088	5.8∙10^−9^	0.36	0.21
**Triglycerides**	HDL2	0.0054	0.00087	6.5∙10^−10^	0.24	0.27
	Serum	−0.0034	0.00087	7.7∙10^−5^	0.16	0.30
	VLDL	−0.0032	0.00087	0.00026	0.37	0.20
	HDL	−0.0034	0.00093	0.00029	0.55	0.11
**Apolipoproteins**	ApoA1	0.0047	0.00093	5.5∙10^−7^	0.033	0.36
	ApoB/ApoA1	−0.0044	0.00085	2.9∙10^−7^	0.00	0.73
**Particle diameters**	LDL	−0.0045	0.00095	2.5∙10^−6^	0.78	0.011
	HDL	0.0035	0.00085	3.9∙10^−5^	0.11	0.33
**Fatty acids**						
Absolute	Unsaturation %	0.0093	0.00095	9.2∙10^−23^	0.58	0.090
	PUFA	0.0053	0.00095	1.9∙10^−8^	0.26	0.26
	Omega-3	0.0081	0.00093	3.7∙10^−18^	0.57	0.096
	DHA	0.0085	0.00093	3.5∙10^−20^	0.75	0.017
	Omega-6	0.0041	0.00096	1.7∙10^−5^	0.57	0.10
Proportions	SAFA %	−0.0075	0.00097	7.4∙10^−15^	0.77	0.012
	MUFA %	−0.0064	0.00090	1.2∙10^−12^	0.75	0.019
	PUFA %	0.0088	0.00092	1.3∙10^−21^	0.59	0.088
	Omega-3 %	0.0098	0.00096	1.2∙10^−24^	0.87	3.5∙10^−4^
	DHA%	0.010	0.00095	1.8∙10^−27^	0.90	6.0∙10^−5^
	Omega-6 %	0.0059	0.00090	4.5∙10^−11^	0.82	0.0044
	LA %	0.0034	0.00091	0.00020	0.81	0.0059
**Fluid balance**	Albumin	0.0049	0.00096	2.7∙10^−7^	0.89	1.4∙10^−4^
**Amino acids**	Glutamine	−0.0039	0.00096	4.4∙10^−5^	0.00	0.96
	Glysine	−0.0036	0.00094	0.00012	0.00	0.38
	Histidine	0.0054	0.00097	2.0∙10^−8^	0.37	0.20
	Valine	0.0047	0.00090	1.3∙10^−7^	0.65	0.058

Significant *P* values are <0.00032. Models were adjusted for age, sex, smoking, diabetes, and body mass index.

ApoA1, apolipoprotein A-I; ApoB, apolipoprotein B; CE, cholesterol ester; DHA, docosahexaenoic acid; FC, free cholesterol; HDL, high-density lipoprotein; IDL, intermediate-density lipoprotein; LA, linoleic acid; MUFA, monounsaturated fatty acid; PUFA, polyunsaturated fatty acid; SAFA, saturated fatty acid; VLDL, very-low-density lipoprotein.

In Health-2000 and Health-2011, we investigated the associations of the number of teeth at baseline and tooth loss during the 11-y period with the follow-up metabolite concentrations. The baseline tooth count predicted alterations in 43 metabolic measures (27%; Appendix Table 3), whereas no associations with tooth loss were observed (Appendix Table 4).

### Lipoproteins

**Figure 2.**
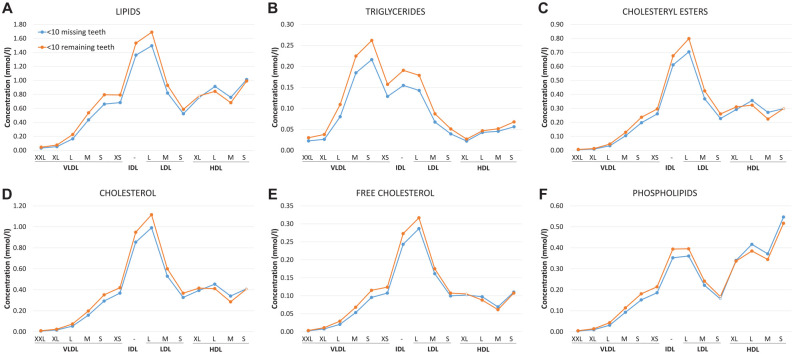
Lipid concentrations of participants with <10 teeth missing (i.e., 23 to 32 teeth remaining; *n* = 3,477) and <10 teeth remaining (*n* = 1,753) in the lipoprotein subgroups in FINRISK-97. (**A–F**) Division into the following subgroups was selected to represent extreme phenotypes of the tooth count: lipids, triglycerides, cholesteryl esters, cholesterol, free cholesterol, and phospholipids. The differences between the groups were calculated by Mann-Whitney *U* test. All differences were significant (*P* < 0.00032) except those indicated with empty circles. HDL, high-density lipoprotein; IDL, intermediate-density lipoprotein; LDL, low-density lipoprotein; VLDL, very-low-density lipoprotein.

Figure 2 represents the lipid concentrations of participants with <10 missing teeth (i.e., 23 to 32 remaining teeth; *n* = 3,477) and those with <10 remaining teeth (*n* = 1,753). Participants with fewer remaining teeth showed higher concentrations of VLDL, intermediate-density lipoprotein (IDL), and LDL lipids. Notably, the most remarkable differences were observed in small VLDL, IDL, and large LDL particles, whereas the divergencies within large VLDL particles (subclasses XXL, XL, and L) remained minor. For HDL, the lipid concentrations appeared equal or marginally lower in the group with <10 remaining teeth than in participants with more teeth. Among the lipid subclasses, the most pronounced differences were observed in triglycerides.

In the meta-analyses (Table 2), the number of teeth was negatively associated with numerous VLDL-related measures, including concentration of XL-sized particles; total lipid and phospholipid contents of subfractions XL, L, and M; and triglycerides of overall and XS VLDL. The number of teeth also showed an inverse association with total and esterified cholesterol in subclasses XXL to M and free cholesterol in XL VLDL. Moreover, there was a negative relationship with serum triglycerides.

**Figure 3.**
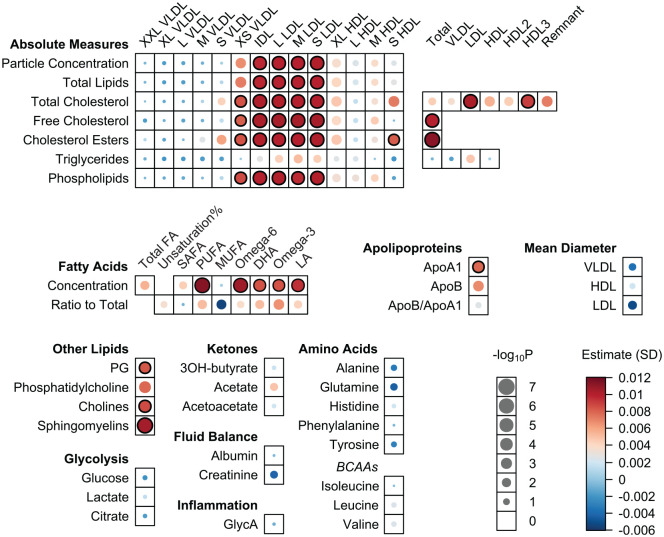
Associations between number of teeth at baseline and metabolite concentrations in an 11-y follow-up in the Health-2000/2011 survey. The analyses were adjusted for baseline metabolite measures, age, sex, smoking status (never, former, current), body mass index, and diabetes. Significant findings (*P* < 0.00032) are indicated with black circles. ApoA1, apolipoprotein A-I; ApoB, apolipoprotein B; BCAA, branched-chain amino acid; DHA, docosahexaenoic acid; FA, fatty acid; GlycA, glycoprotein acetyls; HDL, high-density lipoprotein; IDL, intermediate-density lipoprotein; LA, linoleic acid; LDL, low-density lipoprotein; MUFA, monounsaturated fatty acid; PUFA, polyunsaturated fatty acid; SAFA, saturated fatty acid; VLDL, very-low-density lipoprotein.

Higher number of teeth at baseline was associated with changes in overall, free, and esterified cholesterol and phospholipids of XS VLDL (Fig. 3, Appendix Table 3). The tooth count at baseline was also associated with alterations in particle concentration, total lipid content, cholesterol measurements, and phospholipids of IDL and all LDL subfractions.

Regarding HDL-related measures, the number of teeth presented positive associations with particle concentration, total lipids, cholesterol measures, and phospholipids of subclasses L and M (Fig. 1, Table 2). There was an inverse association between tooth count and triglycerides in S HDL. Additionally, the number of teeth showed positive associations with total triglyceride and cholesterol contents of HDL, along with a larger particle size. The association of the number of teeth with apolipoprotein A-I (ApoA1) was positive, whereas that with the proportion of apolipoprotein B to ApoA1 was negative. The baseline tooth count predicted changes in esterified cholesterol of S HDL as well as in HDL3 cholesterol and ApoA1 (Fig. 3 and Appendix Table 3). Furthermore, the number of teeth at baseline was associated with a change in serum sphingomyelin.

### FAs and Other Metabolites

The number of teeth presented positive associations with unsaturation degree of FAs and absolute and proportional levels of polyunsaturated, omega-6, and omega-3 FAs, including docosahexaenoic acid (DHA; Fig. 1, Table 2). A negative association emerged between tooth count and proportions of monounsaturated FAs (MUFAs) and saturated FAs. A higher number of teeth at baseline predicted changes in concentrations of polyunsaturated, omega-3, and omega-6 FAs, involving DHA and linoleic acid, in follow-up. The *I*^2^ values from the meta-analysis (Table 2, Appendix Table 2) indicated considerable heterogeneity in the associations of several FAs (for DHA and for percentages of DHA, linoleic acid, omega-3, omega-6, and saturated FA; *I*^2^ > 0.75, *P* < 0.10). Nevertheless, when the results from different populations were compared, no major contrasts were observed (Appendix Table 1).

Additionally, we noted positive associations of tooth count with amino acids histidine and valine and negative relationships with glutamine and glycine. The number of teeth also showed a positive association with albumin (Fig. 1).

## Discussion

This study of 3 populations, including an 11-y follow-up and altogether 12,712 participants, indicates that missing teeth are associated with adverse circulating metabolic signatures of an inflammatory nature. The features include high VLDL-related measures and triglyceride concentrations, low HDL-related parameters with a small particle diameter, decreased levels of unsaturated FAs, and high proportions of MUFAs and saturated FAs. These observations are independent of age, sex, smoking, body mass index, and diabetes. Furthermore, missing teeth at baseline predicted changes in multiple lipoprotein- and FA-related measures over the follow-up.

Caries and periodontitis, the most common indications for dental extractions, can mediate systemic inflammation (Sabharwal et al. 2021), which plays a crucial role in the development and progression of cardiometabolic disorders (Furman et al. 2019). During inflammation, serum triglycerides are elevated due to an increased hepatic VLDL production and a decreased clearance of these triglyceride-rich lipoproteins (Feingold and Grunfeld 2000). VLDL is a risk factor for atherosclerotic CVD independent of LDL (Lee et al. 2022). As compared with smaller particles, large VLDL has a stronger association with incident atherosclerosis (Lee et al. 2022) and is also correlated with insulin resistance and diabetes (Wang et al. 2011), whereas a low concentration of large VLDL is considered a predictor for metabolic health (Phillips and Perry 2015). Our results indicate that missing teeth are associated with proatherogenic VLDL particles and total and VLDL triglycerides.

The overall concentration of HDL decreases in inflammatory conditions (Khovidhunkit et al. 2004). Particles undergo remodeling, promoting the production of small HDL, in which ApoA1 is partially replaced by serum amyloid A (Khovidhunkit et al. 2004). The reverse cholesterol efflux capacity of this HDL is impaired (Mutharasan et al. 2017), and it displays diminished anti-inflammatory and antioxidative properties (Feingold and Grunfeld 2000). In addition to changes in the proteome, alterations in the HDL lipidome (e.g., enrichment in triglycerides and sphingomyelin) reduce the cholesterol efflux capacity and impair the anti-inflammatory activity of these particles (Ganjali et al. 2017). Our findings revealed a positive association of tooth count with ApoA1 as well as with concentrations, lipids, and cholesterol contents of L- and M-sized HDL particles. Missing teeth were associated with a smaller HDL diameter and abundant triglycerides in S-HDL and predicted alterations in serum sphingomyelin in the 11-y follow-up. The analysis therefore showed that missing teeth were related to the formation of dysfunctional HDL species with a potentially diminished vasculoprotective capacity.

The observations of the present work are consistent with our previous findings linking periodontitis and caries to metabolic features characterized by inflammation (Salminen et al. 2024). We reported earlier that periodontitis and root canal fillings were associated with a small HDL diameter. In our previous work, signs of periodontitis, caries, and endodontic conditions demonstrated significant negative associations with unsaturated FAs and a strong positive association with the proportion of MUFAs. The present study of missing teeth is in alignment with these findings. The FA profile linked to caries, periodontitis, and now a decreased tooth count manifests proatherogenic characteristics, and the proportion of MUFAs has especially been suggested as a biomarker of systemic inflammation and various disease end points (Julkunen et al. 2023). Our findings on the positive associations between higher tooth count and unsaturated FAs are in line with a previous observation on the relationship between periodontal health and a biomarker of fatty fish consumption (Ottosson et al. 2022). Present observations support the view that missing teeth reflect poor oral health, despite extractions removing local inflammation foci.

Interestingly, branched-chain amino acid valine was found to be associated with periodontal pockets and bleeding on probing (Salminen et al. 2024); however, in the current study, it was linked to a higher number of teeth. Chronic elevations of branched-chain amino acids contribute to the development of insulin resistance and diabetes (Arany and Neinast 2018) but are also associated with a lower risk for numerous conditions, including Alzheimer disease and dementia (Julkunen et al. 2023). The present finding remains ambiguous yet relevant, considering the previous evidence for the relationship between missing teeth and cognitive dysfunction (Asher et al. 2022).

Diet modulates systemic inflammation through anti- and proinflammatory nutrients. Caries is related to high sugar consumption and periodontitis to proinflammatory diet (Syrjäläinen et al. 2024). Missing teeth, albeit replaced by removable partial or complete dentures, impair the masticatory function, affecting eating habits and nutrient intake (Krall et al. 1998). People with poor masticatory performance tend to prefer soft and easy-to-chew foods. Most important, they consume fewer portions of fresh fruits and vegetables (Nowjack-Raymer and Sheiham 2003; Hung et al. 2005). In contrast, mean intakes of saturated FAs and cholesterol increase in relation to declining tooth count (Hung et al. 2005). The quantity of occluding pairs is also associated with all-cause and cardiovascular mortality (Darnaud et al. 2020). Most studies, however, have been conducted among older people, and findings may be poorly generalized to the whole adult population. Additionally, the number of remaining teeth merely corresponds indirectly to occluding pairs and masticatory function. Among the present cohorts, nutritional data were available only from FINRISK-97, and the total intakes of energy, carbohydrate, fat, and protein were not associated with the number of missing teeth (Liljestrand et al. 2015). Nevertheless, diet may influence all of the associations observed in the present study.

Despite the numerous and confirmatory findings that emerged, the study was inconclusive in revealing any effect of tooth loss during the follow-up on changes in metabolic measures. The most feasible explanation for this is the relatively small population size in longitudinal analyses and even smaller quantity of extracted teeth. Of the 1,186 participants in the follow-up, 458 had lost teeth. However, the majority had only 1 or 2 teeth extracted. According to our interpretation, loss of merely a few teeth yields no dramatic effect on the overall systemic inflammatory burden.

Reverse causality may have introduced a bias in the analyses, and numerous confounding factors that could affect the results were unavailable. We adjusted the regression models for diabetes, but other cardiometabolic diseases, such as CVD and hypertension, as well as medications for these conditions could influence the results. We also lacked information on the reasons why study participants had lost teeth, and the associations between different indications for extractions and metabolomics remain an intriguing topic for future studies.

Apart from these limitations, the study has several strengths. It is based on detailed data of 157 metabolites and a large sample size in the cross-sectional analyses. We employed 3 Finnish cohorts, of which 2 were population based and 1 comprised older patients with cardiac indications. Our findings in separate populations were highly aligned and replicated the previously reported direct associations between the number of teeth and HDL cholesterol and indirect associations with triglyceride concentrations (Liljestrand et al. 2015). Thus, our results can be generalized to the Finnish population, although replication studies in diverse populations are required to strengthen the external validity of these findings. The study is a front-runner in exploring the associations between missing teeth and systemic metabolomics on such a large scale.

Our results indicate that missing teeth represent poor oral health and are associated with disadvantageous features of systemic metabolites, characterized by inflammation and numerous risk factors for cardiometabolic diseases. We demonstrate that circulating metabolites may convey the established link between oral inflammation and systemic conditions. The relevance of our findings is highlighted by the prevalence of oral diseases contributing to tooth loss and cardiometabolic diseases.

## Author Contributions

A.M. Halme, contributed to design, data analysis and interpretation, drafted the manuscript; A. Salminen, contributed to design, data acquisition, analysis, and interpretation, drafted the manuscript; A.L. Suominen, P. Mäntylä, S. Männistö, V. Salomaa, contributed to design, data acquisition and interpretation, critically revised the manuscript; A. Havulinna, contributed to acquisition, critically revised the manuscript; K. Buhlin, S. Paju, contributed to acquisition and interpretation, critically revised the manuscript; W. Sattler, contributed to interpretation, critically revised the manuscript; J. Sinisalo, contributed to conception and design, data acquisition, critically revised the manuscript; P.J. Pussinen, contributed to conception and design, data acquisition and interpretation, drafted the manuscript. All authors gave their final approval and agreed to be accountable for all aspects of the work ensuring integrity and accuracy.

## Supplemental Material

sj-docx-1-jdr-10.1177_00220345241298219 – Supplemental material for Metabolic Profiling of Individuals with Missing Teeth and Tooth Loss Supplemental material, sj-docx-1-jdr-10.1177_00220345241298219 for Metabolic Profiling of Individuals with Missing Teeth and Tooth Loss by A.M. Halme, A. Salminen, A.L. Suominen, A. Havulinna, P. Mäntylä, K. Buhlin, S. Paju, S. Männistö, V. Salomaa, W. Sattler, J. Sinisalo and P.J. Pussinen in Journal of Dental Research
