# Supplementary material for: Metabolic Profiling of Individuals with Missing Teeth and Tooth Loss
Source: J Dent Res. 2025 Jan 3;104(4):389–97. doi: 10.1177/00220345241298219 (PMC11909773; doi:10.1177/00220345241298219)
Supplement: sj-docx-1-jdr-10.1177_00220345241298219 – Supplemental material for Metabolic Profiling of Individuals with Missing Teeth and Tooth Loss [file sj-docx-1-jdr-10.1177_00220345241298219.docx]

**­­­­Metabolic profiling of individuals with missing teeth and tooth loss**

Halme AM, Salminen A, Suominen AL, Havulinna A, Mäntylä P, Buhlin K, Paju S, Männistö S, Salomaa V, Sattler W, Sinisalo J, Pussinen PJ

**APPENDIX**

**Appendix Table 1.** Associations between the number of teeth and 157 metabolites in the three cohorts separately.

**Appendix Table 2.** Associations between the number of teeth and all 157 metabolites in the meta-analysis.

**Appendix Table 3.** Associations between the baseline number of teeth and metabolic measures after the 11-year follow-up in the Health-2000 and Health-2011 studies.

**Appendix Table 4.** Associations of tooth loss and 157 metabolic measures in the 11-year follow-up in the Health-2000 and Health-2011 studies.

**Appendix Table 1.** Associations between the number of teeth and 157 metabolites in the three cohorts separately. Linear regression models were adjusted for age, sex, smoking status (current/former/never), body mass index, and diabetes. Significant results (p<0.00032) are indicated in bold.

| **Target** | **Subclass** | **Metabolite** |  | **Parogene** | | |  | **FINRISK-97** | |  |  | **Health-2000** | | |
| --- | --- | --- | --- | --- | --- | --- | --- | --- | --- | --- | --- | --- | --- | --- |
|  |  |  |  | **β** | **SD for β** | **p** |  | **β** | **SD for β** | **p** |  | **β** | **SD for β** | **p** |
| **VLDL** | XXL | Particle concentration |  | -0.013 | 0.0058 | 0.022 |  | -0.0043 | 0.0011 | **0.00021** |  | -0.00063 | 0.0015 | 0.68 |
|  |  | Total lipids |  | -0.013 | 0.0058 | 0.023 |  | -0.0043 | 0.0011 | **0.00019** |  | -0.00066 | 0.0015 | 0.66 |
|  |  | Cholesterol |  | -0.013 | 0.0058 | 0.023 |  | -0.0042 | 0.0011 | **0.00023** |  | -0.0012 | 0.0015 | 0.43 |
|  |  | Free Cholesterol |  | -0.012 | 0.0058 | 0.034 |  | -0.0041 | 0.0012 | 0.00039 |  | -0.00065 | 0.0015 | 0.67 |
|  |  | Cholesterol esters |  | -0.014 | 0.0059 | 0.018 |  | -0.0041 | 0.0011 | **0.00027** |  | -0.0015 | 0.0015 | 0.33 |
|  |  | Triglycerides |  | -0.013 | 0.0058 | 0.022 |  | -0.0043 | 0.0011 | **0.00016** |  | -0.00062 | 0.0015 | 0.68 |
|  |  | Phospholipids |  | -0.012 | 0.0058 | 0.037 |  | -0.0038 | 0.0012 | 0.0011 |  | -0.00049 | 0.0015 | 0.75 |
|  | XL | Particle concentration |  | -0.012 | 0.0058 | 0.039 |  | -0.0049 | 0.0012 | **2.4E-05** |  | -0.00076 | 0.0015 | 0.61 |
|  |  | Total lipids |  | -0.012 | 0.0058 | 0.046 |  | -0.0049 | 0.0011 | **2.2E-05** |  | -0.0010 | 0.0015 | 0.50 |
|  |  | Cholesterol |  | -0.012 | 0.0058 | 0.037 |  | -0.0047 | 0.0011 | **3.4E-05** |  | -0.0011 | 0.0015 | 0.46 |
|  |  | Free Cholesterol |  | -0.012 | 0.0058 | 0.044 |  | -0.0044 | 0.0011 | **0.00010** |  | -0.0010 | 0.0015 | 0.49 |
|  |  | Cholesterol esters |  | -0.012 | 0.0058 | 0.032 |  | -0.0049 | 0.0011 | **1.6E-05** |  | -0.00094 | 0.0015 | 0.54 |
|  |  | Triglycerides |  | -0.012 | 0.0058 | 0.045 |  | -0.0050 | 0.0012 | **1.6E-05** |  | -0.00079 | 0.0015 | 0.60 |
|  |  | Phospholipids |  | -0.012 | 0.0058 | 0.043 |  | -0.0044 | 0.0011 | **0.00014** |  | -0.0011 | 0.0015 | 0.48 |
|  | L | Particle concentration |  | -0.011 | 0.0057 | 0.051 |  | -0.0045 | 0.0011 | **0.00010** |  | -0.00035 | 0.0015 | 0.82 |
|  |  | Total lipids |  | -0.010 | 0.0057 | 0.075 |  | -0.0044 | 0.0011 | **0.00008** |  | -0.0010 | 0.0015 | 0.50 |
|  |  | Cholesterol |  | -0.012 | 0.0058 | 0.037 |  | -0.0046 | 0.0011 | **4.8E-05** |  | -0.00094 | 0.0015 | 0.53 |
|  |  | Free Cholesterol |  | -0.011 | 0.0058 | 0.057 |  | -0.0045 | 0.0011 | **0.00007** |  | -0.00041 | 0.0015 | 0.79 |
|  |  | Cholesterol esters |  | -0.013 | 0.0058 | 0.023 |  | -0.0045 | 0.0011 | **5.8E-05** |  | -0.0011 | 0.0015 | 0.46 |
|  |  | Triglycerides |  | -0.010 | 0.0057 | 0.077 |  | -0.0043 | 0.0011 | **0.00015** |  | -0.00058 | 0.0015 | 0.69 |
|  |  | Phospholipids |  | -0.011 | 0.0057 | 0.056 |  | -0.0044 | 0.0011 | **0.00011** |  | -0.00071 | 0.0015 | 0.63 |
|  | M | Particle concentration |  | -0.011 | 0.0057 | 0.056 |  | -0.0039 | 0.0011 | 0.00048 |  | -0.00072 | 0.0015 | 0.63 |
|  |  | Total lipids |  | -0.011 | 0.0057 | 0.062 |  | -0.0039 | 0.0011 | 0.00044 |  | -0.0015 | 0.0014 | 0.29 |
|  |  | Cholesterol |  | -0.012 | 0.0058 | 0.035 |  | -0.0040 | 0.0011 | 0.00037 |  | -0.0018 | 0.0015 | 0.23 |
|  |  | Free Cholesterol |  | -0.010 | 0.0057 | 0.070 |  | -0.0040 | 0.0011 | **0.00030** |  | -0.00094 | 0.0015 | 0.53 |
|  |  | Cholesterol esters |  | -0.013 | 0.0058 | 0.021 |  | -0.0038 | 0.0011 | 0.00073 |  | -0.0023 | 0.0015 | 0.13 |
|  |  | Triglycerides |  | -0.010 | 0.0057 | 0.079 |  | -0.0037 | 0.0011 | 0.00094 |  | -0.0011 | 0.0015 | 0.44 |
|  |  | Phospholipids |  | -0.011 | 0.0057 | 0.054 |  | -0.0040 | 0.0011 | **0.00030** |  | -0.0013 | 0.0015 | 0.37 |
|  | S | Particle concentration |  | -0.010 | 0.0057 | 0.096 |  | -0.0030 | 0.0011 | 0.0055 |  | -0.0017 | 0.0015 | 0.24 |
|  |  | Total lipids |  | -0.010 | 0.0057 | 0.081 |  | -0.0030 | 0.0011 | 0.0047 |  | -0.0022 | 0.0015 | 0.13 |
|  |  | Cholesterol |  | -0.0073 | 0.0058 | 0.21 |  | -0.0025 | 0.0011 | 0.021 |  | -0.0021 | 0.0015 | 0.17 |
|  |  | Free Cholesterol |  | -0.0079 | 0.0058 | 0.17 |  | -0.0030 | 0.0011 | 0.0067 |  | -0.0019 | 0.0015 | 0.20 |
|  |  | Cholesterol esters |  | -0.0061 | 0.0059 | 0.30 |  | -0.0022 | 0.0011 | 0.046 |  | -0.0022 | 0.0015 | 0.14 |
|  |  | Triglycerides |  | -0.010 | 0.0057 | 0.070 |  | -0.0033 | 0.0011 | 0.0026 |  | -0.0016 | 0.0015 | 0.27 |
|  |  | Phospholipids |  | -0.010 | 0.0058 | 0.094 |  | -0.0030 | 0.0011 | 0.0059 |  | -0.0021 | 0.0015 | 0.15 |
|  | XS | Particle concentration |  | -0.0046 | 0.0058 | 0.43 |  | -0.0027 | 0.0011 | 0.014 |  | -0.0027 | 0.0016 | 0.082 |
|  |  | Total lipids |  | -0.0043 | 0.0059 | 0.47 |  | -0.0026 | 0.0011 | 0.017 |  | -0.0025 | 0.0016 | 0.11 |
|  |  | Cholesterol |  | -0.0020 | 0.0059 | 0.73 |  | -0.0023 | 0.0011 | 0.044 |  | -0.0024 | 0.0016 | 0.14 |
|  |  | Free Cholesterol |  | 0.0014 | 0.0059 | 0.82 |  | -0.0019 | 0.0011 | 0.087 |  | -0.0017 | 0.0016 | 0.28 |
|  |  | Cholesterol esters |  | -0.0034 | 0.0059 | 0.56 |  | -0.0024 | 0.0011 | 0.034 |  | -0.0027 | 0.0016 | 0.09 |
|  |  | Triglycerides |  | -0.0087 | 0.0057 | 0.13 |  | -0.0035 | 0.0011 | 0.0014 |  | -0.0028 | 0.0015 | 0.06 |
|  |  | Phospholipids |  | -0.00039 | 0.0059 | 0.95 |  | -0.0017 | 0.0011 | 0.14 |  | -0.0025 | 0.0016 | 0.12 |
| **IDL** | | Particle concentration |  | 0.0021 | 0.0058 | 0.72 |  | -0.0019 | 0.0011 | 0.098 |  | -0.0028 | 0.0016 | 0.082 |
|  |  | Total lipids |  | 0.0028 | 0.0058 | 0.63 |  | -0.0016 | 0.0011 | 0.17 |  | -0.0023 | 0.0016 | 0.15 |
|  |  | Cholesterol |  | 0.0041 | 0.0058 | 0.49 |  | -0.0010 | 0.0012 | 0.39 |  | -0.0022 | 0.0016 | 0.17 |
|  |  | Free Cholesterol |  | 0.0071 | 0.0058 | 0.22 |  | -0.00081 | 0.0012 | 0.49 |  | -0.0020 | 0.0016 | 0.22 |
|  |  | Cholesterol esters |  | 0.0027 | 0.0058 | 0.65 |  | -0.0011 | 0.0012 | 0.34 |  | -0.0024 | 0.0016 | 0.13 |
|  |  | Triglycerides |  | -0.0050 | 0.0058 | 0.39 |  | -0.0030 | 0.0011 | 0.0065 |  | -0.0041 | 0.0015 | 0.0070 |
|  |  | Phospholipids |  | 0.0045 | 0.0058 | 0.44 |  | -0.0017 | 0.0012 | 0.15 |  | -0.0028 | 0.0016 | 0.08 |
| **LDL** | L | Particle concentration |  | 0.0060 | 0.0059 | 0.31 |  | -0.0013 | 0.0011 | 0.24 |  | -0.0024 | 0.0016 | 0.14 |
|  |  | Total lipids |  | 0.0070 | 0.0059 | 0.24 |  | -0.0010 | 0.0011 | 0.37 |  | -0.0019 | 0.0016 | 0.24 |
|  |  | Cholesterol |  | 0.0067 | 0.0059 | 0.26 |  | -0.00056 | 0.0012 | 0.63 |  | -0.0018 | 0.0016 | 0.27 |
|  |  | Free Cholesterol |  | 0.0086 | 0.0058 | 0.14 |  | -0.00067 | 0.0012 | 0.57 |  | -0.0021 | 0.0016 | 0.19 |
|  |  | Cholesterol esters |  | 0.0057 | 0.0059 | 0.33 |  | -0.00057 | 0.0012 | 0.62 |  | -0.0018 | 0.0016 | 0.26 |
|  |  | Triglycerides |  | 0.0012 | 0.0058 | 0.83 |  | -0.0030 | 0.0011 | 0.0065 |  | -0.0035 | 0.0015 | 0.023 |
|  |  | Phospholipids |  | 0.0074 | 0.0059 | 0.21 |  | -0.0010 | 0.0012 | 0.39 |  | -0.0021 | 0.0016 | 0.20 |
|  | M | Particle concentration |  | 0.0086 | 0.0059 | 0.14 |  | -0.00070 | 0.0011 | 0.54 |  | -0.0022 | 0.0016 | 0.18 |
|  |  | Total lipids |  | 0.0094 | 0.0059 | 0.11 |  | -0.00045 | 0.0011 | 0.69 |  | -0.0018 | 0.0016 | 0.26 |
|  |  | Cholesterol |  | 0.0085 | 0.0059 | 0.15 |  | 6.9E-05 | 0.0012 | 0.95 |  | -0.0017 | 0.0016 | 0.29 |
|  |  | Free Cholesterol |  | 0.012 | 0.0058 | 0.04 |  | -0.00048 | 0.0012 | 0.68 |  | -0.0017 | 0.0016 | 0.30 |
|  |  | Cholesterol esters |  | 0.0073 | 0.0058 | 0.21 |  | 0.00017 | 0.0012 | 0.89 |  | -0.0017 | 0.0016 | 0.27 |
|  |  | Triglycerides |  | 0.0068 | 0.0059 | 0.25 |  | -0.0026 | 0.0011 | 0.021 |  | -0.0034 | 0.0015 | 0.028 |
|  |  | Phospholipids |  | 0.0094 | 0.0059 | 0.11 |  | -0.00080 | 0.0012 | 0.49 |  | -0.0019 | 0.0016 | 0.22 |
|  | S | Particle concentration |  | 0.011 | 0.0058 | 0.052 |  | -0.00023 | 0.0012 | 0.84 |  | -0.0015 | 0.0016 | 0.33 |
|  |  | Total lipids |  | 0.012 | 0.0058 | 0.036 |  | -2.2E-05 | 0.0012 | 0.99 |  | -0.0013 | 0.0016 | 0.41 |
|  |  | Cholesterol |  | 0.011 | 0.0058 | 0.063 |  | 0.00045 | 0.0012 | 0.70 |  | -0.0014 | 0.0016 | 0.37 |
|  |  | Free Cholesterol |  | 0.016 | 0.0058 | 0.0079 |  | -0.00010 | 0.0012 | 0.93 |  | -0.0010 | 0.0016 | 0.53 |
|  |  | Cholesterol esters |  | 0.0093 | 0.0058 | 0.11 |  | 0.00055 | 0.0012 | 0.64 |  | -0.0016 | 0.0016 | 0.32 |
|  |  | Triglycerides |  | 0.0047 | 0.0059 | 0.43 |  | -0.0029 | 0.0011 | 0.011 |  | -0.0022 | 0.0015 | 0.15 |
|  |  | Phospholipids |  | 0.015 | 0.0058 | 0.013 |  | 0.00025 | 0.0012 | 0.83 |  | -0.00093 | 0.0016 | 0.56 |
| **HDL** | XL | Particle concentration |  | 0.0079 | 0.0054 | 0.15 |  | 0.0014 | 0.0011 | 0.21 |  | 0.0050 | 0.0014 | 0.00033 |
|  |  | Total lipids |  | 0.0077 | 0.0054 | 0.16 |  | 0.0014 | 0.0011 | 0.23 |  | 0.0043 | 0.0014 | 0.0018 |
|  |  | Cholesterol |  | 0.010 | 0.0055 | 0.077 |  | 0.00058 | 0.0012 | 0.62 |  | 0.0050 | 0.0014 | 0.00043 |
|  |  | Free Cholesterol |  | 0.010 | 0.0054 | 0.065 |  | 0.00022 | 0.0011 | 0.85 |  | 0.0044 | 0.0014 | 0.0018 |
|  |  | Cholesterol esters |  | 0.010 | 0.0056 | 0.086 |  | 0.00070 | 0.0012 | 0.55 |  | 0.0055 | 0.0014 | **0.00015** |
|  |  | Triglycerides |  | -0.0083 | 0.0059 | 0.16 |  | -0.0047 | 0.0012 | **0.00010** |  | 0.0012 | 0.0016 | 0.47 |
|  |  | Phospholipids |  | 0.0068 | 0.0054 | 0.21 |  | 0.0025 | 0.0011 | 0.020 |  | 0.0041 | 0.0014 | 0.0023 |
|  | L | Particle concentration |  | 0.010 | 0.0054 | 0.053 |  | 0.0044 | 0.0011 | **5.6E-05** |  | 0.0047 | 0.0014 | 0.00091 |
|  |  | Total lipids |  | 0.010 | 0.0054 | 0.059 |  | 0.0045 | 0.0011 | **3.8E-05** |  | 0.0042 | 0.0014 | 0.0032 |
|  |  | Cholesterol |  | 0.011 | 0.0053 | 0.044 |  | 0.0045 | 0.0011 | **3.1E-05** |  | 0.0048 | 0.0014 | 0.00056 |
|  |  | Free Cholesterol |  | 0.012 | 0.0053 | 0.028 |  | 0.0045 | 0.0011 | **3.1E-05** |  | 0.0053 | 0.0014 | **0.00015** |
|  |  | Cholesterol esters |  | 0.011 | 0.0054 | 0.049 |  | 0.0045 | 0.0011 | **3.6E-05** |  | 0.0048 | 0.0014 | 0.00057 |
|  |  | Triglycerides |  | -0.0029 | 0.0055 | 0.59 |  | -0.00056 | 0.0012 | 0.64 |  | 0.0017 | 0.0015 | 0.28 |
|  |  | Phospholipids |  | 0.011 | 0.0054 | 0.047 |  | 0.0047 | 0.0011 | **1.3E-05** |  | 0.0041 | 0.0014 | 0.0041 |
|  | M | Particle concentration |  | 0.0060 | 0.0057 | 0.29 |  | 0.0051 | 0.0012 | **2.3E-05** |  | 0.0027 | 0.0016 | 0.092 |
|  |  | Total lipids |  | 0.0061 | 0.0057 | 0.28 |  | 0.0052 | 0.0012 | **1.5E-05** |  | 0.0028 | 0.0016 | 0.081 |
|  |  | Cholesterol |  | 0.0081 | 0.0057 | 0.16 |  | 0.0056 | 0.0012 | **2.4E-06** |  | 0.0034 | 0.0016 | 0.031 |
|  |  | Free Cholesterol |  | 0.010 | 0.0056 | 0.081 |  | 0.0053 | 0.0012 | **8.3E-06** |  | 0.0028 | 0.0016 | 0.080 |
|  |  | Cholesterol esters |  | 0.0077 | 0.0058 | 0.18 |  | 0.0055 | 0.0012 | **2.4E-06** |  | 0.0036 | 0.0016 | 0.024 |
|  |  | Triglycerides |  | -0.016 | 0.0058 | 0.0070 |  | -0.0018 | 0.0012 | 0.14 |  | -0.0023 | 0.0015 | 0.14 |
|  |  | Phospholipids |  | 0.0064 | 0.0057 | 0.26 |  | 0.0048 | 0.0012 | **6.8E-05** |  | 0.0025 | 0.0016 | 0.12 |
|  | S | Particle concentration |  | 0.0079 | 0.0058 | 0.17 |  | 0.0011 | 0.0013 | 0.40 |  | -0.00074 | 0.0016 | 0.65 |
|  |  | Total lipids |  | 0.0088 | 0.0059 | 0.14 |  | 0.0016 | 0.0013 | 0.21 |  | -0.00072 | 0.0016 | 0.66 |
|  |  | Cholesterol |  | 0.016 | 0.0059 | 0.0056 |  | 0.0027 | 0.0013 | 0.029 |  | -0.00093 | 0.0016 | 0.57 |
|  |  | Free Cholesterol |  | 0.0088 | 0.0059 | 0.13 |  | 0.00016 | 0.0012 | 0.90 |  | 0.00075 | 0.0016 | 0.64 |
|  |  | Cholesterol esters |  | 0.017 | 0.0059 | 0.0050 |  | 0.0032 | 0.0012 | 0.011 |  | -0.0013 | 0.0016 | 0.43 |
|  |  | Triglycerides |  | -0.011 | 0.0057 | 0.047 |  | -0.0051 | 0.0011 | **6.4E-06** |  | -0.0046 | 0.0015 | 0.0019 |
|  |  | Phospholipids |  | 0.0031 | 0.0059 | 0.59 |  | 0.0018 | 0.0012 | 0.13 |  | 0.00045 | 0.0016 | 0.78 |
| **Cholesterol** | | Serum |  | 0.0082 | 0.0058 | 0.16 |  | 0.00045 | 0.0012 | 0.70 |  | 5.3E-05 | 0.0016 | 0.97 |
|  |  | VLDL |  | -0.011 | 0.0058 | 0.069 |  | -0.0034 | 0.0011 | 0.0016 |  | -0.0022 | 0.0015 | 0.14 |
|  |  | LDL |  | 0.0081 | 0.0059 | 0.17 |  | -0.00010 | 0.0012 | 0.93 |  | -0.0015 | 0.0016 | 0.36 |
|  |  | HDL |  | 0.014 | 0.0054 | 0.0080 |  | 0.0051 | 0.0011 | **5.5E-06** |  | 0.0045 | 0.0015 | 0.0024 |
|  |  | HDL2 |  | 0.014 | 0.0054 | 0.011 |  | 0.0054 | 0.0011 | **8.1E-07** |  | 0.0046 | 0.0015 | 0.0016 |
|  |  | HDL3 |  | 0.015 | 0.0056 | 0.0069 |  | -0.00081 | 0.0012 | 0.51 |  | 0.0025 | 0.0016 | 0.11 |
|  |  | Free |  | 0.0068 | 0.0059 | 0.24 |  | -0.00065 | 0.0012 | 0.58 |  | -0.00051 | 0.0016 | 0.75 |
|  |  | Esterified |  | 0.0086 | 0.0059 | 0.14 |  | 0.00087 | 0.0012 | 0.46 |  | 4.6E-05 | 0.0016 | 0.98 |
|  |  | Remnant |  | -0.0055 | 0.0059 | 0.35 |  | -0.0026 | 0.0011 | 0.021 |  | -0.0020 | 0.0015 | 0.20 |
| **Triglycerides** | | Serum |  | -0.010 | 0.0057 | 0.076 |  | -0.0039 | 0.0011 | 0.00036 |  | -0.0021 | 0.0015 | 0.15 |
|  |  | VLDL |  | -0.010 | 0.0056 | 0.093 |  | -0.0039 | 0.0011 | 0.00036 |  | -0.0014 | 0.0015 | 0.33 |
|  |  | LDL |  | -0.0023 | 0.0058 | 0.69 |  | -0.0029 | 0.0011 | 0.0082 |  | -0.0035 | 0.0015 | 0.025 |
|  |  | HDL |  | -0.014 | 0.0059 | 0.021 |  | -0.0039 | 0.0012 | 0.00082 |  | -0.0016 | 0.0016 | 0.31 |
| **Apolipoproteins** | | ApoA1 |  | 0.012 | 0.0055 | 0.025 |  | 0.0045 | 0.0012 | **0.00012** |  | 0.0043 | 0.0016 | 0.0073 |
|  |  | ApoB |  | -0.0037 | 0.0059 | 0.53 |  | -0.0023 | 0.0011 | 0.040 |  | -0.0017 | 0.0015 | 0.26 |
|  |  | ApoB/ApoA1 |  | -0.0086 | 0.0056 | 0.13 |  | -0.0041 | 0.0011 | **0.00014** |  | -0.0045 | 0.0014 | 0.0015 |
| **Particle diameters** | | VLDL |  | -0.0084 | 0.0056 | 0.14 |  | -0.0038 | 0.0011 | 0.00083 |  | -0.0015 | 0.0015 | 0.31 |
|  |  | LDL |  | -0.022 | 0.0058 | 0.00020 |  | -0.0039 | 0.0012 | 0.0014 |  | -0.0041 | 0.0015 | 0.0078 |
|  |  | HDL |  | 0.0073 | 0.0053 | 0.17 |  | 0.0025 | 0.0011 | 0.026 |  | 0.0048 | 0.0014 | 0.00045 |
| **Other lipids** | | Phosphatidyl- glycerol |  | 0.0024 | 0.0058 | 0.68 |  | 0.0015 | 0.0012 | 0.22 |  | 0.0015 | 0.0016 | 0.37 |
|  |  | Phosphatidyl- choline |  | -0.00031 | 0.0058 | 0.96 |  | 0.0015 | 0.0012 | 0.21 |  | 0.0011 | 0.0016 | 0.50 |
|  |  | Total cholines |  | 0.0034 | 0.0058 | 0.56 |  | 0.0017 | 0.0012 | 0.16 |  | 0.0013 | 0.0016 | 0.41 |
|  |  | Sphingomyelin |  | 0.014 | 0.0057 | 0.013 |  | 0.0032 | 0.0012 | 0.0064 |  | 0.00077 | 0.0016 | 0.63 |
| **Fatty adids** | Absolute | Total fatty acids |  | -0.0029 | 0.0060 | 0.63 |  | 0.00095 | 0.0012 | 0.42 |  | 0.00025 | 0.0016 | 0.88 |
|  |  | Unsaturation% |  | 0.017 | 0.0059 | 0.0038 |  | 0.0078 | 0.0012 | **2.1E-10** |  | 0.011 | 0.0016 | **5.4E-13** |
|  |  | SAFA |  | -0.0043 | 0.0060 | 0.47 |  | -0.0015 | 0.0012 | 0.20 |  | -0.00034 | 0.0016 | 0.83 |
|  |  | MUFA |  | -0.011 | 0.0059 | 0.070 |  | -0.0012 | 0.0011 | 0.28 |  | -0.0021 | 0.0015 | 0.16 |
|  |  | PUFA |  | 0.0078 | 0.0059 | 0.19 |  | 0.0064 | 0.0012 | **8.8E-08** |  | 0.0032 | 0.0016 | 0.048 |
|  |  | Omega-3 |  | 0.013 | 0.0060 | 0.032 |  | 0.0066 | 0.0012 | **1.7E-08** |  | 0.011 | 0.0016 | **4.3E-11** |
|  |  | DHA |  | 0.015 | 0.0060 | 0.011 |  | 0.0066 | 0.0012 | **1.7E-08** |  | 0.012 | 0.0016 | **1.9E-13** |
|  |  | Omega-6 |  | 0.0055 | 0.0059 | 0.35 |  | 0.0056 | 0.0012 | **3.5E-06** |  | 0.0013 | 0.0016 | 0.42 |
|  |  | LA |  | 0.0034 | 0.0060 | 0.56 |  | 0.0048 | 0.0012 | **9.5E-05** |  | 2.2E-05 | 0.0016 | 0.99 |
|  | Proportions | SAFA% |  | -0.0047 | 0.0060 | 0.44 |  | -0.0098 | 0.0012 | **2.6E-15** |  | -0.0039 | 0.0016 | 0.016 |
|  |  | MUFA% |  | -0.020 | 0.0056 | 0.00043 |  | -0.0051 | 0.0011 | **8.7E-06** |  | -0.0077 | 0.0015 | **3.4E-07** |
|  |  | PUFA% |  | 0.019 | 0.0057 | 0.00069 |  | 0.0093 | 0.0012 | **4.0E-15** |  | 0.0072 | 0.0015 | **2.2E-06** |
|  |  | Omega-3% |  | 0.021 | 0.0059 | 0.00048 |  | 0.0069 | 0.0012 | **1.5E-08** |  | 0.014 | 0.0016 | **3.0E-18** |
|  |  | DHA% |  | 0.023 | 0.0059 | 0.00011 |  | 0.0072 | 0.0012 | **3.1E-09** |  | 0.015 | 0.0016 | **1.7E-20** |
|  |  | Omega-6% |  | 0.015 | 0.0057 | 0.010 |  | 0.0078 | 0.0012 | **2.1E-11** |  | 0.0023 | 0.0015 | 0.12 |
|  |  | LA% |  | 0.0094 | 0.0058 | 0.11 |  | 0.0054 | 0.0012 | **3.8E-06** |  | -0.00038 | 0.0015 | 0.80 |
| **Glycolysis** | | Glucose |  | 0.010 | 0.0056 | 0.086 |  | 0.00015 | 0.0011 | 0.90 |  | 0.00092 | 0.0015 | 0.54 |
|  |  | Lactate |  | -0.00037 | 0.0058 | 0.95 |  | -0.00081 | 0.0012 | 0.50 |  | -0.0010 | 0.0015 | 0.53 |
|  |  | Citrate |  | 0.0081 | 0.0059 | 0.17 |  | -0.0026 | 0.0012 | 0.03 |  | -0.0043 | 0.0015 | 0.0038 |
|  |  | Glycerol |  | -0.0030 | 0.0061 | 0.62 |  | -0.0010 | 0.0012 | 0.40 |  | -0.0014 | 0.0017 | 0.40 |
| **Ketone bodies** | | Pyruvate |  | -0.013 | 0.0059 | 0.027 |  | -0.0014 | 0.0012 | 0.28 |  | 0.00042 | 0.0015 | 0.78 |
|  |  | Beta- hydroxybutyrate |  | 0.0061 | 0.0058 | 0.30 |  | 0.0040 | 0.0012 | 0.0014 |  | -0.0026 | 0.0016 | 0.11 |
|  |  | Acetate |  | 0.0010 | 0.0059 | 0.87 |  | 0.0030 | 0.0012 | 0.013 |  | 0.0039 | 0.0016 | 0.017 |
|  |  | Acetoacetate |  | 0.0059 | 0.0060 | 0.32 |  | 0.0045 | 0.0012 | **0.00024** |  | 0.00065 | 0.0016 | 0.69 |
| **Fluid balance** | | Albumin |  | -0.00066 | 0.0059 | 0.91 |  | 0.0081 | 0.0012 | **3.2E-11** |  | -0.00020 | 0.0016 | 0.90 |
|  |  | Creatinine |  | 0.0014 | 0.0058 | 0.81 |  | 0.0013 | 0.0011 | 0.21 |  | 0.0043 | 0.0015 | 0.0032 |
| **Inflammation** | | Glycoprotein acetyls |  | -0.021 | 0.0055 | 0.00014 |  | -0.0025 | 0.0011 | 0.025 |  | -0.0018 | 0.0015 | 0.24 |
| **Amino acids** |  | Alanine |  | -0.0069 | 0.0059 | 0.24 |  | 0.0019 | 0.0012 | 0.10 |  | 0.0030 | 0.0016 | 0.051 |
|  |  | Glutamine |  | -0.0047 | 0.0060 | 0.43 |  | -0.0041 | 0.0012 | 0.00085 |  | -0.0036 | 0.0016 | 0.025 |
|  |  | Glysine |  | -0.0051 | 0.0056 | 0.37 |  | -0.0026 | 0.0012 | 0.032 |  | -0.0053 | 0.0016 | 0.00076 |
|  |  | Histidine |  | 0.010 | 0.0058 | 0.10 |  | 0.0065 | 0.0012 | **1.4E-07** |  | 0.0032 | 0.0016 | 0.042 |
|  |  | Phenylalanine |  | -0.0034 | 0.0058 | 0.56 |  | 0.00074 | 0.0011 | 0.50 |  | -0.0041 | 0.0015 | 0.0080 |
|  |  | Tyrosine |  | 0.0043 | 0.0058 | 0.46 |  | 0.0038 | 0.0012 | 0.0014 |  | 0.0015 | 0.0015 | 0.33 |
|  | Branched chain | Isoleucine |  | -0.0079 | 0.0056 | 0.16 |  | 0.00090 | 0.0011 | 0.43 |  | -7.6E-05 | 0.0015 | 0.96 |
|  |  | Leucine |  | 0.0026 | 0.0056 | 0.65 |  | 0.0026 | 0.0011 | 0.020 |  | 0.00012 | 0.0015 | 0.93 |
|  |  | Valine |  | 0.0020 | 0.0055 | 0.71 |  | 0.0064 | 0.0011 | **1.9E-08** |  | 0.0020 | 0.0015 | 0.19 |

**Appendix Table 2.** Associations between the number of teeth and all 157 metabolites in the meta-analysis**.** Linear regression models were adjusted for age, sex, smoking (current/former/never), body mass index, and diabetes. The results from the three cohorts were combined by a fixed-effects meta-analysis. Significant results (p<0.00032) are indicated in bold.

| **Target** | **Subclass** | **Metabolite** | **Meta-analysis** | | | **Heterogeneity** | |
| --- | --- | --- | --- | --- | --- | --- | --- |
|  |  |  | **β** | **SD for β** | **p** | **I^2^** | **p** |
| **VLDL** | XXL | Particle concentration | -0.0032 | 0.00090 | 0.00042 | 0.71 | 0.034 |
|  |  | Total lipids | -0.0032 | 0.00090 | 0.00038 | 0.70 | 0.035 |
|  |  | Cholesterol | -0.0034 | 0.00090 | **0.00019** | 0.63 | 0.065 |
|  |  | Free Cholesterol | -0.0031 | 0.00091 | 0.00073 | 0.66 | 0.052 |
|  |  | Cholesterol esters | -0.0034 | 0.00090 | **0.00013** | 0.61 | 0.075 |
|  |  | Triglycerides | -0.0032 | 0.00091 | 0.00034 | 0.71 | 0.031 |
|  |  | Phospholipids | -0.0028 | 0.00091 | 0.0020 | 0.64 | 0.061 |
|  | XL | Particle concentration | -0.0036 | 0.00090 | **8.2E-05** | 0.71 | 0.032 |
|  |  | Total lipids | -0.0036 | 0.00090 | **5.2E-05** | 0.67 | 0.047 |
|  |  | Cholesterol | -0.0036 | 0.00090 | **5.5E-05** | 0.66 | 0.054 |
|  |  | Free Cholesterol | -0.0034 | 0.00090 | **0.00015** | 0.62 | 0.070 |
|  |  | Cholesterol esters | -0.0037 | 0.00090 | **4.1E-05** | 0.70 | 0.034 |
|  |  | Triglycerides | -0.0036 | 0.00090 | **6.2E-05** | 0.71 | 0.032 |
|  |  | Phospholipids | -0.0034 | 0.00090 | **0.00019** | 0.62 | 0.073 |
|  | L | Particle concentration | -0.0031 | 0.00089 | 0.00046 | 0.71 | 0.033 |
|  |  | Total lipids | -0.0033 | 0.00088 | **0.00016** | 0.59 | 0.085 |
|  |  | Cholesterol | -0.0035 | 0.00089 | **9.5E-05** | 0.67 | 0.047 |
|  |  | Free Cholesterol | -0.0032 | 0.00090 | 0.00035 | 0.70 | 0.036 |
|  |  | Cholesterol esters | -0.0035 | 0.00089 | **7.6E-05** | 0.68 | 0.043 |
|  |  | Triglycerides | -0.0031 | 0.00089 | 0.00049 | 0.64 | 0.061 |
|  |  | Phospholipids | -0.0032 | 0.00089 | **0.00028** | 0.65 | 0.055 |
|  | M | Particle concentration | -0.0029 | 0.00088 | 0.00084 | 0.60 | 0.083 |
|  |  | Total lipids | -0.0032 | 0.00087 | **0.00023** | 0.42 | 0.18 |
|  |  | Cholesterol | -0.0034 | 0.00088 | **0.00012** | 0.47 | 0.15 |
|  |  | Free Cholesterol | -0.0031 | 0.00088 | 0.00045 | 0.55 | 0.11 |
|  |  | Cholesterol esters | -0.0035 | 0.00088 | **8.4E-05** | 0.46 | 0.16 |
|  |  | Triglycerides | -0.0029 | 0.00087 | 0.00086 | 0.44 | 0.17 |
|  |  | Phospholipids | -0.0032 | 0.00087 | **0.00023** | 0.51 | 0.13 |
|  | S | Particle concentration | -0.0027 | 0.00087 | 0.0016 | 0.00 | 0.38 |
|  |  | Total lipids | -0.0029 | 0.00086 | 0.00067 | 0.00 | 0.41 |
|  |  | Cholesterol | -0.0025 | 0.00087 | 0.0044 | 0.00 | 0.68 |
|  |  | Free Cholesterol | -0.0027 | 0.00087 | 0.0018 | 0.00 | 0.56 |
|  |  | Cholesterol esters | -0.0023 | 0.00088 | 0.0094 | 0.00 | 0.81 |
|  |  | Triglycerides | -0.0029 | 0.00087 | 0.00093 | 0.23 | 0.27 |
|  |  | Phospholipids | -0.0029 | 0.00087 | 0.0010 | 0.00 | 0.44 |
|  | XS | Particle concentration | -0.0028 | 0.00089 | 0.0020 | 0.00 | 0.95 |
|  |  | Total lipids | -0.0026 | 0.00089 | 0.0030 | 0.00 | 0.96 |
|  |  | Cholesterol | -0.0023 | 0.00090 | 0.011 | 0.00 | 1.00 |
|  |  | Free Cholesterol | -0.0018 | 0.00091 | 0.050 | 0.00 | 0.86 |
|  |  | Cholesterol esters | -0.0025 | 0.00090 | 0.0054 | 0.00 | 0.97 |
|  |  | Triglycerides | -0.0034 | 0.00088 | **0.00011** | 0.00 | 0.60 |
|  |  | Phospholipids | -0.0019 | 0.00091 | 0.035 | 0.00 | 0.90 |
| **IDL** | | Particle concentration | -0.0021 | 0.00092 | 0.023 | 0.00 | 0.70 |
|  |  | Total lipids | -0.0017 | 0.00092 | 0.063 | 0.00 | 0.69 |
|  |  | Cholesterol | -0.0013 | 0.00094 | 0.17 | 0.00 | 0.54 |
|  |  | Free Cholesterol | -0.0010 | 0.00094 | 0.29 | 0.15 | 0.31 |
|  |  | Cholesterol esters | -0.0015 | 0.00094 | 0.12 | 0.00 | 0.63 |
|  |  | Triglycerides | -0.0035 | 0.00089 | **0.00011** | 0.00 | 0.82 |
|  |  | Phospholipids | -0.0019 | 0.00093 | 0.042 | 0.00 | 0.46 |
| **LDL** | L | Particle concentration | -0.0015 | 0.00092 | 0.10 | 0.00 | 0.38 |
|  |  | Total lipids | -0.0011 | 0.00092 | 0.22 | 0.065 | 0.34 |
|  |  | Cholesterol | -0.00079 | 0.00093 | 0.40 | 0.021 | 0.36 |
|  |  | Free Cholesterol | -0.00091 | 0.00094 | 0.33 | 0.39 | 0.20 |
|  |  | Cholesterol esters | -0.00083 | 0.00093 | 0.37 | 0.00 | 0.43 |
|  |  | Triglycerides | -0.0031 | 0.00090 | 0.00053 | 0.00 | 0.73 |
|  |  | Phospholipids | -0.0011 | 0.00093 | 0.22 | 0.19 | 0.29 |
|  | M | Particle concentration | -0.0010 | 0.00092 | 0.30 | 0.39 | 0.19 |
|  |  | Total lipids | -0.00065 | 0.00092 | 0.48 | 0.43 | 0.18 |
|  |  | Cholesterol | -0.00031 | 0.00093 | 0.74 | 0.36 | 0.21 |
|  |  | Free Cholesterol | -0.00056 | 0.00093 | 0.55 | 0.61 | 0.076 |
|  |  | Cholesterol esters | -0.00031 | 0.00093 | 0.74 | 0.25 | 0.26 |
|  |  | Triglycerides | -0.0027 | 0.00090 | 0.0032 | 0.29 | 0.24 |
|  |  | Phospholipids | -0.00093 | 0.00092 | 0.31 | 0.44 | 0.17 |
|  | S | Particle concentration | -0.00038 | 0.00092 | 0.68 | 0.56 | 0.10 |
|  |  | Total lipids | -0.00015 | 0.00092 | 0.87 | 0.61 | 0.079 |
|  |  | Cholesterol | 0.00008 | 0.00094 | 0.94 | 0.55 | 0.11 |
|  |  | Free Cholesterol | -2.2E-06 | 0.00094 | 1.00 | 0.73 | 0.023 |
|  |  | Cholesterol esters | 3.9E-05 | 0.00094 | 0.97 | 0.47 | 0.15 |
|  |  | Triglycerides | -0.0025 | 0.00090 | 0.0061 | 0.00 | 0.45 |
|  |  | Phospholipids | 0.00021 | 0.00094 | 0.82 | 0.69 | 0.038 |
| **HDL** | XL | Particle concentration | 0.0030 | 0.00087 | 0.00060 | 0.59 | 0.088 |
|  |  | Total lipids | 0.0027 | 0.00087 | 0.0020 | 0.45 | 0.16 |
|  |  | Cholesterol | 0.0026 | 0.00089 | 0.0042 | 0.74 | 0.022 |
|  |  | Free Cholesterol | 0.0021 | 0.00088 | 0.017 | 0.73 | 0.024 |
|  |  | Cholesterol esters | 0.0028 | 0.00090 | 0.0020 | 0.75 | 0.017 |
|  |  | Triglycerides | -0.0027 | 0.00095 | 0.0040 | 0.79 | 0.0093 |
|  |  | Phospholipids | 0.0033 | 0.00084 | **0.00010** | 0.00 | 0.53 |
|  | L | Particle concentration | 0.0047 | 0.00085 | **4.8E-08** | 0.00 | 0.55 |
|  |  | Total lipids | 0.0045 | 0.00085 | **1.1E-07** | 0.00 | 0.56 |
|  |  | Cholesterol | 0.0048 | 0.00084 | **1.5E-08** | 0.00 | 0.51 |
|  |  | Free Cholesterol | 0.0050 | 0.00084 | **3.6E-09** | 0.00 | 0.39 |
|  |  | Cholesterol esters | 0.0048 | 0.00084 | **1.9E-08** | 0.00 | 0.54 |
|  |  | Triglycerides | 0.00020 | 0.00094 | 0.83 | 0.00 | 0.44 |
|  |  | Phospholipids | 0.0047 | 0.00085 | **4.8E-08** | 0.00 | 0.49 |
|  | M | Particle concentration | 0.0043 | 0.00095 | **6.4E-06** | 0.00 | 0.46 |
|  |  | Total lipids | 0.0044 | 0.00095 | **3.6E-06** | 0.00 | 0.46 |
|  |  | Cholesterol | 0.0049 | 0.00093 | **1.6E-07** | 0.00 | 0.48 |
|  |  | Free Cholesterol | 0.0046 | 0.00094 | **1.3E-06** | 0.22 | 0.28 |
|  |  | Cholesterol esters | 0.0049 | 0.00093 | **1.2E-07** | 0.00 | 0.54 |
|  |  | Triglycerides | -0.0023 | 0.00093 | 0.013 | 0.64 | 0.062 |
|  |  | Phospholipids | 0.0040 | 0.00095 | **2.1E-05** | 0.00 | 0.46 |
|  | S | Particle concentration | 0.00059 | 0.00097 | 0.55 | 0.17 | 0.30 |
|  |  | Total lipids | 0.00093 | 0.00097 | 0.34 | 0.35 | 0.21 |
|  |  | Cholesterol | 0.0018 | 0.00098 | 0.068 | 0.79 | 0.0085 |
|  |  | Free Cholesterol | 0.00061 | 0.00098 | 0.53 | 0.048 | 0.35 |
|  |  | Cholesterol esters | 0.0019 | 0.00097 | 0.049 | 0.82 | 0.0038 |
|  |  | Triglycerides | -0.0051 | 0.00089 | **1.1E-08** | 0.00 | 0.52 |
|  |  | Phospholipids | 0.0014 | 0.00096 | 0.15 | 0.00 | 0.75 |
| **Cholesterol** | | Serum | 0.00052 | 0.00094 | 0.58 | 0.00 | 0.40 |
|  |  | VLDL | -0.0032 | 0.00086 | **0.00026** | 0.048 | 0.35 |
|  |  | LDL | -0.00036 | 0.00093 | 0.70 | 0.24 | 0.27 |
|  |  | HDL | 0.0051 | 0.00088 | **5.8E-09** | 0.36 | 0.21 |
|  |  | HDL2 | 0.0054 | 0.00087 | **6.5E-10** | 0.24 | 0.27 |
|  |  | HDL3 | 0.00088 | 0.00096 | 0.36 | 0.79 | 0.0085 |
|  |  | Free | -0.00041 | 0.00093 | 0.66 | 0.00 | 0.46 |
|  |  | Esterified | 0.00079 | 0.00094 | 0.40 | 2.1E-04 | 0.37 |
|  |  | Remnant | -0.0024 | 0.00089 | 0.0061 | 0.00 | 0.84 |
| **Triglycerides** | | Serum | -0.0034 | 0.00087 | **7.7E-05** | 0.16 | 0.30 |
|  |  | VLDL | -0.0032 | 0.00087 | **0.00026** | 0.37 | 0.20 |
|  |  | LDL | -0.0031 | 0.00089 | 0.00051 | 0.00 | 0.95 |
|  |  | HDL | -0.0034 | 0.00093 | **0.00029** | 0.55 | 0.11 |
| **Apolipoproteins** | | ApoA1 | 0.0047 | 0.00093 | **5.5E-07** | 0.033 | 0.36 |
|  |  | ApoB | -0.0021 | 0.00089 | 0.017 | 0.00 | 0.92 |
|  |  | ApoB/ApoA1 | -0.0044 | 0.00085 | **2.9E-07** | 0.00 | 0.73 |
| **Particle diameters** | | VLDL | -0.0030 | 0.00088 | 0.00055 | 0.20 | 0.29 |
|  |  | LDL | -0.0045 | 0.00095 | **2.5E-06** | 0.78 | 0.011 |
|  |  | HDL | 0.0035 | 0.00085 | **3.9E-05** | 0.11 | 0.33 |
| **Other lipids** | | Phosphatidyl-glycerol | 0.0015 | 0.00095 | 0.11 | 0.00 | 0.99 |
|  |  | Phosphatidyl- choline | 0.0013 | 0.00095 | 0.17 | 0.00 | 0.94 |
|  |  | Total cholines | 0.0016 | 0.00095 | 0.088 | 0.00 | 0.94 |
|  |  | Sphingomyelin | 0.0027 | 0.00094 | 0.0043 | 0.65 | 0.057 |
| **Fatty adids** | Absolute | Total fatty acids | 0.00061 | 0.00093 | 0.51 | 0.00 | 0.79 |
|  |  | Unsaturation% | 0.0093 | 0.00095 | **9.2E-23** | 0.58 | 0.090 |
|  |  | SAFA | -0.0012 | 0.00093 | 0.21 | 0.00 | 0.73 |
|  |  | MUFA | -0.0018 | 0.00091 | 0.050 | 0.22 | 0.28 |
|  |  | PUFA | 0.0053 | 0.00095 | **1.9E-08** | 0.26 | 0.26 |
|  |  | Omega-3 | 0.0081 | 0.00093 | **3.7E-18** | 0.57 | 0.096 |
|  |  | DHA | 0.0085 | 0.00093 | **3.5E-20** | 0.75 | 0.017 |
|  |  | Omega-6 | 0.0041 | 0.00096 | **1.7E-05** | 0.57 | 0.10 |
|  |  | LA | 0.0031 | 0.00097 | 0.0015 | 0.63 | 0.065 |
|  | Proportions | SAFA% | -0.0075 | 0.00097 | **7.4E-15** | 0.77 | 0.012 |
|  |  | MUFA% | -0.0064 | 0.00090 | **1.2E-12** | 0.75 | 0.019 |
|  |  | PUFA% | 0.0088 | 0.00092 | **1.3E-21** | 0.59 | 0.088 |
|  |  | Omega-3% | 0.0098 | 0.00096 | **1.2E-24** | 0.87 | 3.5E-04 |
|  |  | DHA% | 0.010 | 0.00095 | **1.8E-27** | 0.90 | 6.0E-05 |
|  |  | Omega-6% | 0.0059 | 0.00090 | **4.5E-11** | 0.82 | 0.0044 |
|  |  | LA% | 0.0034 | 0.00091 | **0.00020** | 0.81 | 0.0059 |
| **Glycolysis** | | Glucose | 0.00065 | 0.00089 | 0.46 | 0.29 | 0.25 |
|  |  | Lactate | -0.00086 | 0.00093 | 0.35 | 0.00 | 0.99 |
|  |  | Citrate | -0.0030 | 0.00093 | 0.0013 | 0.55 | 0.11 |
|  |  | Glycerol | -0.0012 | 0.00098 | 0.21 | 0.00 | 0.94 |
| **Ketone bodies** | | Pyruvate | -0.0010 | 0.00095 | 0.31 | 0.61 | 0.076 |
|  |  | Beta- hydroxybutyrate | 0.0017 | 0.00097 | 0.087 | 0.82 | 0.0041 |
|  |  | Acetate | 0.0032 | 0.00096 | 0.00070 | 0.00 | 0.85 |
|  |  | Acetoacetate | 0.0032 | 0.00096 | 0.00096 | 0.47 | 0.15 |
| **Fluid balance** | | Albumin | 0.0049 | 0.00096 | **2.7E-07** | 0.89 | 1.4E-04 |
|  |  | Creatinine | 0.0023 | 0.00085 | 0.0059 | 0.27 | 0.25 |
| **Inflammation** | | Glycoprotein acetyls | -0.0027 | 0.00088 | 0.0020 | 0.83 | 0.0031 |
| **Amino acids** |  | Alanine | 0.0021 | 0.00091 | 0.024 | 0.27 | 0.25 |
|  |  | Glutamine | -0.0039 | 0.00096 | **4.4E-05** | 0.00 | 0.96 |
|  |  | Glysine | -0.0036 | 0.00094 | **0.00012** | 0.00 | 0.38 |
|  |  | Histidine | 0.0054 | 0.00097 | **2.0E-08** | 0.37 | 0.20 |
|  |  | Phenylalanine | -0.0010 | 0.00089 | 0.28 | 0.70 | 0.036 |
|  |  | Tyrosine | 0.0030 | 0.00093 | 0.0014 | 0.00 | 0.48 |
|  | Branched chain | Isoleucine | 0.00032 | 0.00089 | 0.72 | 0.20 | 0.29 |
|  |  | Leucine | 0.0017 | 0.00088 | 0.051 | 0.00 | 0.40 |
|  |  | Valine | 0.0047 | 0.00090 | **1.3E-07** | 0.65 | 0.058 |

**Appendix Table 3.** Associations between the baseline number of teeth and metabolic measures after the 11-year follow-up in the Health-2000 and Health-2011 studies. Linear regression models were adjusted for baseline metabolite concentrations, age, sex, smoking status (current/former/never), body mass index, and diabetes. Significant results (p<0.00032) are indicated in bold.

| **Target** | **Subclass** | **Metabolite** | **β** | **SD for β** | **p** |
| --- | --- | --- | --- | --- | --- |
| **VLDL** | XXL | Particle concentration | -0.00066 | 0.0024 | 0.78 |
|  |  | Total lipids | -0.00064 | 0.0024 | 0.79 |
|  |  | Cholesterol | -0.00033 | 0.0024 | 0.89 |
|  |  | Free cholesterol | -0.0017 | 0.0024 | 0.49 |
|  |  | Cholesterol esters | 0.00097 | 0.0024 | 0.69 |
|  |  | Triglycerides | -0.00095 | 0.0024 | 0.69 |
|  |  | Phospholipids | -0.00051 | 0.0024 | 0.83 |
|  | XL | Particle concentration | -0.0016 | 0.0025 | 0.51 |
|  |  | Total lipids | -0.0016 | 0.0025 | 0.51 |
|  |  | Cholesterol | -0.0011 | 0.0025 | 0.68 |
|  |  | Free cholesterol | -0.00082 | 0.0026 | 0.75 |
|  |  | Cholesterol esters | -0.0013 | 0.0025 | 0.60 |
|  |  | Triglycerides | -0.0019 | 0.0024 | 0.43 |
|  |  | Phospholipids | -0.0010 | 0.0025 | 0.68 |
|  | L | Particle concentration | -0.0014 | 0.0022 | 0.53 |
|  |  | Total lipids | -0.0015 | 0.0022 | 0.48 |
|  |  | Cholesterol | -0.00090 | 0.0022 | 0.69 |
|  |  | Free cholesterol | -0.0014 | 0.0022 | 0.54 |
|  |  | Cholesterol esters | -0.00053 | 0.0022 | 0.81 |
|  |  | Triglycerides | -0.0018 | 0.0022 | 0.42 |
|  |  | Phospholipids | -0.0011 | 0.0022 | 0.63 |
|  | M | Particle concentration | -0.00080 | 0.0019 | 0.68 |
|  |  | Total lipids | -0.00087 | 0.0019 | 0.64 |
|  |  | Cholesterol | 0.00082 | 0.0020 | 0.68 |
|  |  | Free cholesterol | -0.00085 | 0.0019 | 0.66 |
|  |  | Cholesterol esters | 0.0026 | 0.0021 | 0.21 |
|  |  | Triglycerides | -0.0016 | 0.0018 | 0.37 |
|  |  | Phospholipids | -0.00052 | 0.0019 | 0.79 |
|  | S | Particle concentration | 0.00060 | 0.0019 | 0.76 |
|  |  | Total lipids | 0.00098 | 0.0019 | 0.61 |
|  |  | Cholesterol | 0.0043 | 0.0021 | 0.04 |
|  |  | Free cholesterol | 0.0014 | 0.0020 | 0.48 |
|  |  | Cholesterol esters | 0.0060 | 0.0022 | 0.01 |
|  |  | Triglycerides | -0.0015 | 0.0019 | 0.41 |
|  |  | Phospholipids | 0.0012 | 0.0020 | 0.54 |
|  | XS | Particle concentration | 0.0066 | 0.0022 | 0.0023 |
|  |  | Total lipids | 0.0071 | 0.0022 | 0.0011 |
|  |  | Cholesterol | 0.0084 | 0.0022 | **0.00013** |
|  |  | Free cholesterol | 0.0080 | 0.0022 | **0.00026** |
|  |  | Cholesterol esters | 0.0084 | 0.0022 | **0.00013** |
|  |  | Triglycerides | 0.00016 | 0.0019 | 0.93 |
|  |  | Phospholipids | 0.0086 | 0.0022 | **8.7E-05** |
| **IDL** | | Particle concentration | 0.0095 | 0.0022 | **1.4E-05** |
|  |  | Total lipids | 0.0096 | 0.0022 | **1.2E-05** |
|  |  | Cholesterol | 0.010 | 0.0022 | **4.4E-06** |
|  |  | Free cholesterol | 0.010 | 0.0022 | **2.3E-06** |
|  |  | Cholesterol esters | 0.0099 | 0.0022 | **7.2E-06** |
|  |  | Triglycerides | 0.0028 | 0.0020 | 0.17 |
|  |  | Phospholipids | 0.0099 | 0.0022 | **5.8E-06** |
| **LDL** | L | Particle concentration | 0.0098 | 0.0022 | **8.0E-06** |
|  |  | Total lipids | 0.0097 | 0.0022 | **1.0E-05** |
|  |  | Cholesterol | 0.0099 | 0.0022 | **6.1E-06** |
|  |  | Free cholesterol | 0.010 | 0.0022 | **2.5E-06** |
|  |  | Cholesterol esters | 0.0099 | 0.0022 | **7.9E-06** |
|  |  | Triglycerides | 0.0044 | 0.0021 | 0.032 |
|  |  | Phospholipids | 0.0099 | 0.0022 | **6.4E-06** |
|  | M | Particle concentration | 0.0098 | 0.0022 | **8.0E-06** |
|  |  | Total lipids | 0.0097 | 0.0022 | **1.0E-05** |
|  |  | Cholesterol | 0.0099 | 0.0022 | **6.7E-06** |
|  |  | Free cholesterol | 0.010 | 0.0022 | **3.5E-06** |
|  |  | Cholesterol esters | 0.0099 | 0.0022 | **7.4E-06** |
|  |  | Triglycerides | 0.0051 | 0.0021 | 0.013 |
|  |  | Phospholipids | 0.0094 | 0.0022 | **2.0E-05** |
|  | S | Particle concentration | 0.0099 | 0.0022 | **6.3E-06** |
|  |  | Total lipids | 0.0098 | 0.0022 | **7.9E-06** |
|  |  | Cholesterol | 0.0099 | 0.0022 | **6.6E-06** |
|  |  | Free cholesterol | 0.010 | 0.0022 | **3.0E-06** |
|  |  | Cholesterol esters | 0.010 | 0.0022 | **4.9E-06** |
|  |  | Triglycerides | 0.0045 | 0.0021 | 0.033 |
|  |  | Phospholipids | 0.0099 | 0.0022 | **6.2E-06** |
| **HDL** | XL | Particle concentration | 0.0037 | 0.0015 | 0.014 |
|  |  | Total lipids | 0.0041 | 0.0015 | 0.0056 |
|  |  | Cholesterol | 0.0047 | 0.0016 | 0.0026 |
|  |  | Free cholesterol | 0.0041 | 0.0015 | 0.0081 |
|  |  | Cholesterol esters | 0.0048 | 0.0016 | 0.0031 |
|  |  | Triglycerides | 0.0026 | 0.0022 | 0.24 |
|  |  | Phospholipids | 0.0034 | 0.0015 | 0.021 |
|  | L | Particle concentration | 0.0025 | 0.0018 | 0.16 |
|  |  | Total lipids | 0.0022 | 0.0017 | 0.20 |
|  |  | Cholesterol | 0.0015 | 0.0017 | 0.37 |
|  |  | Free cholesterol | 0.0016 | 0.0016 | 0.31 |
|  |  | Cholesterol esters | 0.0015 | 0.0017 | 0.37 |
|  |  | Triglycerides | 0.0024 | 0.0020 | 0.24 |
|  |  | Phospholipids | 0.0032 | 0.0018 | 0.08 |
|  | M | Particle concentration | 0.0039 | 0.0022 | 0.074 |
|  |  | Total lipids | 0.0039 | 0.0022 | 0.078 |
|  |  | Cholesterol | 0.0035 | 0.0022 | 0.11 |
|  |  | Free cholesterol | 0.0046 | 0.0022 | 0.034 |
|  |  | Cholesterol esters | 0.0032 | 0.0022 | 0.14 |
|  |  | Triglycerides | -7.1E-06 | 0.0020 | 1.00 |
|  |  | Phospholipids | 0.0043 | 0.0022 | 0.05 |
|  | S | Particle concentration | 0.0025 | 0.0022 | 0.25 |
|  |  | Total lipids | 0.0028 | 0.0022 | 0.20 |
|  |  | Cholesterol | 0.0072 | 0.0022 | 0.0015 |
|  |  | Free cholesterol | -0.00036 | 0.0022 | 0.87 |
|  |  | Cholesterol esters | 0.0081 | 0.0022 | **3.0E-04** |
|  |  | Triglycerides | -0.0019 | 0.0019 | 0.32 |
|  |  | Phospholipids | -0.0014 | 0.0021 | 0.53 |
| **Cholesterol** | | Serum | 0.011 | 0.0022 | **1.2E-06** |
|  |  | VLDL | 0.0036 | 0.0021 | 0.086 |
|  |  | LDL | 0.010 | 0.0022 | **3.7E-06** |
|  |  | HDL | 0.0054 | 0.0018 | 0.0025 |
|  |  | HDL2 | 0.0045 | 0.0018 | 0.011 |
|  |  | HDL3 | 0.0089 | 0.0020 | **9.9E-06** |
|  |  | Free | 0.0097 | 0.0022 | **7.8E-06** |
|  |  | Esterified | 0.011 | 0.0022 | **5.5E-07** |
|  |  | Remnant | 0.0071 | 0.0022 | 0.0013 |
| **Triglycerides** | | Serum | -0.00078 | 0.0019 | 0.68 |
|  |  | VLDL | -0.0015 | 0.0018 | 0.42 |
|  |  | LDL | 0.0048 | 0.0021 | 0.020 |
|  |  | HDL | 0.00066 | 0.0022 | 0.76 |
| **Apolipoproteins** | | ApoA1 | 0.0077 | 0.0020 | **0.00016** |
|  |  | ApoB | 0.0068 | 0.0022 | 0.0018 |
|  |  | ApoB/ApoA1 | 0.0026 | 0.0019 | 0.16 |
| **Particle diameters** | | VLDL | -0.0035 | 0.0018 | 0.055 |
|  |  | LDL | -0.0049 | 0.0020 | 0.014 |
|  |  | HDL | 0.0020 | 0.0014 | 0.17 |
| **Other lipids** | | Phosphatidylglycerol | 0.0081 | 0.0022 | **0.00018** |
|  |  | Phosphatidylcholine | 0.0077 | 0.0022 | 0.00036 |
|  |  | Total cholines | 0.0085 | 0.0021 | **5.6E-05** |
|  |  | Sphingomyelin | 0.010 | 0.0020 | **2.5E-07** |
| **Fatty adids** | Absolute | Total fatty acids | 0.0055 | 0.0022 | 0.014 |
|  |  | Unsaturation% | 0.0035 | 0.0021 | 0.092 |
|  |  | SAFA | 0.0045 | 0.0022 | 0.042 |
|  |  | MUFA | 0.00057 | 0.0021 | 0.786 |
|  |  | PUFA | 0.011 | 0.0022 | **4.7E-07** |
|  |  | Omega-3 | 0.0084 | 0.0021 | **0.00010** |
|  |  | DHA | 0.0084 | 0.0021 | **6.9E-05** |
|  |  | Omega-6 | 0.011 | 0.0022 | **2.1E-06** |
|  |  | LA | 0.0094 | 0.0022 | **2.5E-05** |
|  | Proportions | SAFA% | -0.00072 | 0.0022 | 0.75 |
|  |  | MUFA% | -0.0052 | 0.0019 | 0.0048 |
|  |  | PUFA% | 0.0051 | 0.0020 | 0.0088 |
|  |  | Omega-3% | 0.0066 | 0.0021 | 0.0019 |
|  |  | DHA% | 0.0051 | 0.0020 | 0.012 |
|  |  | Omega-6% | 0.0038 | 0.0020 | 0.056 |
|  |  | LA% | 0.0043 | 0.0020 | 0.029 |
| **Glycolysis** | | Glucose | -0.0021 | 0.0022 | 0.33 |
|  |  | Lactate | 0.0015 | 0.0021 | 0.48 |
|  |  | Citrate | -0.0017 | 0.0021 | 0.43 |
|  |  | Glycerol | -0.0030 | 0.0024 | 0.22 |
| **Ketone bodies** | | Pyruvate | 0.00027 | 0.0020 | 0.89 |
|  |  | Beta-hydroxybutyrate | 0.0018 | 0.0023 | 0.43 |
|  |  | Acetate | 0.0049 | 0.0023 | 0.034 |
|  |  | Acetoacetate | 0.0020 | 0.0023 | 0.40 |
| **Fluid balance** | | Albumin | -0.00062 | 0.0023 | 0.79 |
|  |  | Creatinine | -0.0040 | 0.0020 | 0.04 |
| **Inflammation** | | Glycoprotein acetyls | -0.00091 | 0.0021 | 0.67 |
| **Amino acids** |  | Alanine | -0.0031 | 0.0021 | 0.14 |
|  |  | Glutamine | -0.0043 | 0.0023 | 0.057 |
|  |  | Glysine | -0.0023 | 0.0019 | 0.23 |
|  |  | Histidine | 0.0020 | 0.0023 | 0.40 |
|  |  | Phenylalanine | -0.00056 | 0.0022 | 0.80 |
|  |  | Tyrosine | -0.0029 | 0.0022 | 0.19 |
|  | Branched chain | Isoleucine | -0.00036 | 0.0021 | 0.86 |
|  |  | Leucine | 0.0024 | 0.0021 | 0.25 |
|  |  | Valine | 0.0024 | 0.0021 | 0.25 |

**Appendix Table 4.** Associations of tooth loss and 157 metabolic measures in the 11-year follow-up in the Health-2000 and Health-2011 cohorts. We calculated linear regression models between the tooth loss in the follow-up as numeric and binomial (no teeth loss or ≥1 tooth lost) and metabolites after the follow-up. The models were adjusted for the baseline metabolite concentrations and number of teeth, age, sex, smoking status (current/former/never), body mass index, and diabetes. Suggestively significant results (p<0.05) are indicated in bold.

|  |  |  |  | **Tooth loss as numeric** | | |  | **Tooth loss as binomial** | | |
| --- | --- | --- | --- | --- | --- | --- | --- | --- | --- | --- |
| **Target** | **Subclass** | **Metabolite** |  | **β** | **SD for β** | **p** |  | **β** | **SD for β** | **p** |
| **VLDL** | XXL | Particle concentration |  | -0.023 | 0.015 | 0.13 |  | -0.011 | 0.065 | 0.87 |
|  |  | Total lipids |  | -0.023 | 0.015 | 0.12 |  | -0.013 | 0.065 | 0.84 |
|  |  | Cholesterol |  | -0.028 | 0.015 | 0.059 |  | -0.030 | 0.064 | 0.64 |
|  |  | Free cholesterol |  | -0.021 | 0.015 | 0.16 |  | -0.0081 | 0.065 | 0.90 |
|  |  | Cholesterol esters |  | -0.033 | 0.015 | **0.026** |  | -0.047 | 0.065 | 0.47 |
|  |  | Triglycerides |  | -0.022 | 0.015 | 0.14 |  | -0.010 | 0.065 | 0.88 |
|  |  | Phospholipids |  | -0.023 | 0.015 | 0.12 |  | -0.0054 | 0.065 | 0.93 |
|  | XL | Particle concentration |  | -0.019 | 0.015 | 0.22 |  | -0.025 | 0.067 | 0.71 |
|  |  | Total lipids |  | -0.018 | 0.015 | 0.24 |  | -0.027 | 0.067 | 0.68 |
|  |  | Cholesterol |  | -0.023 | 0.015 | 0.13 |  | -0.043 | 0.068 | 0.53 |
|  |  | Free cholesterol |  | -0.023 | 0.016 | 0.15 |  | -0.029 | 0.069 | 0.68 |
|  |  | Cholesterol esters |  | -0.023 | 0.015 | 0.13 |  | -0.051 | 0.068 | 0.45 |
|  |  | Triglycerides |  | -0.017 | 0.015 | 0.27 |  | -0.025 | 0.066 | 0.70 |
|  |  | Phospholipids |  | -0.021 | 0.016 | 0.18 |  | -0.033 | 0.068 | 0.63 |
|  | L | Particle concentration |  | -0.020 | 0.013 | 0.14 |  | -0.049 | 0.059 | 0.41 |
|  |  | Total lipids |  | -0.017 | 0.013 | 0.17 |  | -0.054 | 0.058 | 0.35 |
|  |  | Cholesterol |  | -0.021 | 0.013 | 0.11 |  | -0.056 | 0.059 | 0.35 |
|  |  | Free cholesterol |  | -0.020 | 0.013 | 0.14 |  | -0.041 | 0.060 | 0.49 |
|  |  | Cholesterol esters |  | -0.021 | 0.013 | 0.12 |  | -0.066 | 0.059 | 0.26 |
|  |  | Triglycerides |  | -0.017 | 0.013 | 0.18 |  | -0.052 | 0.058 | 0.37 |
|  |  | Phospholipids |  | -0.019 | 0.013 | 0.14 |  | -0.047 | 0.059 | 0.43 |
|  | M | Particle concentration |  | -0.014 | 0.012 | 0.25 |  | -0.022 | 0.051 | 0.66 |
|  |  | Total lipids |  | -0.011 | 0.012 | 0.37 |  | -0.015 | 0.050 | 0.77 |
|  |  | Cholesterol |  | -0.016 | 0.013 | 0.19 |  | -0.031 | 0.054 | 0.56 |
|  |  | Free cholesterol |  | -0.013 | 0.012 | 0.27 |  | -0.019 | 0.052 | 0.71 |
|  |  | Cholesterol esters |  | -0.019 | 0.013 | 0.14 |  | -0.035 | 0.056 | 0.53 |
|  |  | Triglycerides |  | -0.010 | 0.012 | 0.37 |  | -0.019 | 0.050 | 0.70 |
|  |  | Phospholipids |  | -0.014 | 0.012 | 0.25 |  | -0.019 | 0.051 | 0.71 |
|  | S | Particle concentration |  | -0.020 | 0.012 | 0.10 |  | -0.053 | 0.053 | 0.32 |
|  |  | Total lipids |  | -0.018 | 0.012 | 0.14 |  | -0.041 | 0.052 | 0.44 |
|  |  | Cholesterol |  | -0.021 | 0.013 | 0.12 |  | -0.022 | 0.057 | 0.71 |
|  |  | Free cholesterol |  | -0.020 | 0.013 | 0.12 |  | -0.047 | 0.054 | 0.39 |
|  |  | Cholesterol esters |  | -0.023 | 0.014 | 0.10 |  | -0.015 | 0.059 | 0.80 |
|  |  | Triglycerides |  | -0.015 | 0.012 | 0.21 |  | -0.048 | 0.050 | 0.34 |
|  |  | Phospholipids |  | -0.020 | 0.012 | 0.10 |  | -0.054 | 0.053 | 0.31 |
|  | XS | Particle concentration |  | -0.018 | 0.013 | 0.18 |  | 0.00019 | 0.058 | 1.00 |
|  |  | Total lipids |  | -0.017 | 0.013 | 0.20 |  | 0.0071 | 0.058 | 0.90 |
|  |  | Cholesterol |  | -0.018 | 0.014 | 0.18 |  | 0.013 | 0.058 | 0.82 |
|  |  | Free cholesterol |  | -0.019 | 0.014 | 0.16 |  | -0.00056 | 0.058 | 0.99 |
|  |  | Cholesterol esters |  | -0.017 | 0.014 | 0.20 |  | 0.018 | 0.058 | 0.75 |
|  |  | Triglycerides |  | -0.014 | 0.012 | 0.27 |  | -0.040 | 0.052 | 0.44 |
|  |  | Phospholipids |  | -0.019 | 0.014 | 0.16 |  | 0.0091 | 0.058 | 0.88 |
| **IDL** | | Particle concentration |  | -0.018 | 0.014 | 0.18 |  | 0.027 | 0.058 | 0.64 |
|  |  | Total lipids |  | -0.017 | 0.014 | 0.21 |  | 0.031 | 0.058 | 0.60 |
|  |  | Cholesterol |  | -0.021 | 0.014 | 0.12 |  | 0.018 | 0.059 | 0.76 |
|  |  | Free cholesterol |  | -0.020 | 0.013 | 0.13 |  | 0.012 | 0.058 | 0.83 |
|  |  | Cholesterol esters |  | -0.021 | 0.014 | 0.12 |  | 0.020 | 0.059 | 0.73 |
|  |  | Triglycerides |  | -0.009 | 0.012 | 0.48 |  | -0.014 | 0.053 | 0.79 |
|  |  | Phospholipids |  | -0.020 | 0.014 | 0.13 |  | 0.013 | 0.058 | 0.83 |
| **LDL** | L | Particle concentration |  | -0.022 | 0.014 | 0.11 |  | 0.018 | 0.059 | 0.76 |
|  |  | Total lipids |  | -0.021 | 0.014 | 0.13 |  | 0.020 | 0.059 | 0.73 |
|  |  | Cholesterol |  | -0.022 | 0.014 | 0.11 |  | 0.019 | 0.059 | 0.74 |
|  |  | Free cholesterol |  | -0.020 | 0.013 | 0.13 |  | 0.017 | 0.058 | 0.77 |
|  |  | Cholesterol esters |  | -0.022 | 0.014 | 0.10 |  | 0.020 | 0.059 | 0.74 |
|  |  | Triglycerides |  | -0.0071 | 0.013 | 0.57 |  | 0.011 | 0.054 | 0.84 |
|  |  | Phospholipids |  | -0.021 | 0.014 | 0.12 |  | 0.018 | 0.059 | 0.75 |
|  | M | Particle concentration |  | -0.024 | 0.014 | 0.076 |  | 0.010 | 0.059 | 0.86 |
|  |  | Total lipids |  | -0.023 | 0.014 | 0.090 |  | 0.013 | 0.059 | 0.83 |
|  |  | Cholesterol |  | -0.024 | 0.014 | 0.079 |  | 0.010 | 0.059 | 0.86 |
|  |  | Free cholesterol |  | -0.023 | 0.014 | 0.093 |  | 0.015 | 0.059 | 0.80 |
|  |  | Cholesterol esters |  | -0.025 | 0.014 | 0.074 |  | 0.0087 | 0.059 | 0.88 |
|  |  | Triglycerides |  | -0.012 | 0.013 | 0.34 |  | 0.0015 | 0.054 | 0.98 |
|  |  | Phospholipids |  | -0.023 | 0.014 | 0.10 |  | 0.017 | 0.059 | 0.77 |
|  | S | Particle concentration |  | -0.026 | 0.014 | 0.058 |  | -0.00048 | 0.059 | 0.99 |
|  |  | Total lipids |  | -0.025 | 0.014 | 0.067 |  | 0.00057 | 0.058 | 0.99 |
|  |  | Cholesterol |  | -0.025 | 0.014 | 0.066 |  | -0.0016 | 0.058 | 0.98 |
|  |  | Free cholesterol |  | -0.024 | 0.014 | 0.079 |  | -0.0017 | 0.058 | 0.98 |
|  |  | Cholesterol esters |  | -0.026 | 0.014 | 0.058 |  | 0.0021 | 0.059 | 0.97 |
|  |  | Triglycerides |  | -0.021 | 0.013 | 0.11 |  | -0.022 | 0.056 | 0.69 |
|  |  | Phospholipids |  | -0.024 | 0.014 | 0.077 |  | 0.0044 | 0.058 | 0.94 |
| **HDL** | XL | Particle concentration |  | -0.009 | 0.0092 | 0.31 |  | -0.018 | 0.040 | 0.65 |
|  |  | Total lipids |  | -0.011 | 0.0092 | 0.25 |  | -0.016 | 0.039 | 0.69 |
|  |  | Cholesterol |  | -0.012 | 0.010 | 0.23 |  | -0.019 | 0.041 | 0.64 |
|  |  | Free cholesterol |  | -0.0061 | 0.0094 | 0.52 |  | -0.010 | 0.040 | 0.80 |
|  |  | Cholesterol esters |  | -0.013 | 0.010 | 0.17 |  | -0.023 | 0.042 | 0.58 |
|  |  | Triglycerides |  | -0.017 | 0.014 | 0.22 |  | 0.016 | 0.058 | 0.78 |
|  |  | Phospholipids |  | -0.0071 | 0.0091 | 0.44 |  | -0.017 | 0.039 | 0.67 |
|  | L | Particle concentration |  | -0.0073 | 0.011 | 0.51 |  | 0.0018 | 0.045 | 0.97 |
|  |  | Total lipids |  | -0.0078 | 0.011 | 0.47 |  | 0.0038 | 0.045 | 0.93 |
|  |  | Cholesterol |  | -0.0072 | 0.010 | 0.49 |  | -0.00013 | 0.043 | 1.00 |
|  |  | Free cholesterol |  | -0.0077 | 0.010 | 0.46 |  | -0.0071 | 0.043 | 0.87 |
|  |  | Cholesterol esters |  | -0.0068 | 0.010 | 0.51 |  | 0.0010 | 0.043 | 0.98 |
|  |  | Triglycerides |  | -0.0037 | 0.013 | 0.77 |  | 0.011 | 0.052 | 0.83 |
|  |  | Phospholipids |  | -0.0075 | 0.011 | 0.51 |  | 0.0082 | 0.047 | 0.86 |
|  | M | Particle concentration |  | 0.00038 | 0.014 | 0.98 |  | 0.030 | 0.057 | 0.60 |
|  |  | Total lipids |  | 0.00030 | 0.014 | 0.98 |  | 0.031 | 0.057 | 0.59 |
|  |  | Cholesterol |  | -0.00076 | 0.014 | 0.96 |  | 0.037 | 0.057 | 0.52 |
|  |  | Free cholesterol |  | -0.00032 | 0.013 | 0.98 |  | 0.028 | 0.056 | 0.62 |
|  |  | Cholesterol esters |  | -0.0010 | 0.014 | 0.94 |  | 0.039 | 0.057 | 0.50 |
|  |  | Triglycerides |  | -0.0027 | 0.013 | 0.84 |  | -0.015 | 0.055 | 0.78 |
|  |  | Phospholipids |  | 0.0013 | 0.013 | 0.92 |  | 0.026 | 0.056 | 0.65 |
|  | S | Particle concentration |  | -0.00052 | 0.013 | 0.97 |  | 0.0029 | 0.058 | 0.96 |
|  |  | Total lipids |  | -0.00071 | 0.014 | 0.96 |  | 0.0023 | 0.058 | 0.97 |
|  |  | Cholesterol |  | -0.011 | 0.014 | 0.41 |  | -0.016 | 0.060 | 0.79 |
|  |  | Free cholesterol |  | 0.0063 | 0.014 | 0.65 |  | 0.023 | 0.059 | 0.70 |
|  |  | Cholesterol esters |  | -0.014 | 0.014 | 0.29 |  | -0.025 | 0.059 | 0.68 |
|  |  | Triglycerides |  | -0.0013 | 0.012 | 0.91 |  | -0.023 | 0.051 | 0.65 |
|  |  | Phospholipids |  | 0.0076 | 0.013 | 0.57 |  | 0.020 | 0.057 | 0.73 |
| **Cholesterol** | | Serum |  | -0.021 | 0.014 | 0.12 |  | 0.024 | 0.058 | 0.68 |
|  |  | VLDL |  | -0.019 | 0.013 | 0.15 |  | -0.015 | 0.056 | 0.79 |
|  |  | LDL |  | -0.019 | 0.014 | 0.17 |  | 0.027 | 0.058 | 0.65 |
|  |  | HDL |  | -0.0060 | 0.011 | 0.59 |  | 0.0054 | 0.047 | 0.91 |
|  |  | HDL2 |  | -0.0058 | 0.011 | 0.60 |  | 0.014 | 0.047 | 0.77 |
|  |  | HDL3 |  | -0.013 | 0.012 | 0.28 |  | -0.025 | 0.052 | 0.64 |
|  |  | Free |  | -0.020 | 0.013 | 0.14 |  | 0.011 | 0.058 | 0.85 |
|  |  | Esterified |  | -0.022 | 0.014 | 0.10 |  | 0.023 | 0.058 | 0.70 |
|  |  | Remnant |  | -0.019 | 0.014 | 0.16 |  | 0.016 | 0.059 | 0.78 |
| **Triglycerides** | | Serum |  | -0.010 | 0.012 | 0.41 |  | -0.014 | 0.051 | 0.78 |
|  |  | VLDL |  | -0.0094 | 0.012 | 0.42 |  | -0.017 | 0.050 | 0.74 |
|  |  | LDL |  | -0.013 | 0.013 | 0.32 |  | -0.0073 | 0.054 | 0.89 |
|  |  | HDL |  | -0.0064 | 0.014 | 0.64 |  | -0.020 | 0.058 | 0.73 |
| **Apolipoproteins** | | ApoA-I |  | -0.017 | 0.012 | 0.18 |  | 0.013 | 0.053 | 0.81 |
|  |  | ApoB |  | -0.021 | 0.014 | 0.12 |  | 0.010 | 0.058 | 0.86 |
|  |  | ApoB/ApoA-I |  | -0.013 | 0.012 | 0.30 |  | -0.0010 | 0.051 | 0.98 |
| **Particle diameters** | | VLDL |  | 0.00067 | 0.011 | 0.95 |  | -0.0020 | 0.049 | 0.97 |
|  |  | LDL |  | 0.019 | 0.012 | 0.12 |  | 0.054 | 0.053 | 0.30 |
|  |  | HDL |  | -0.0054 | 0.0089 | 0.54 |  | -0.0066 | 0.038 | 0.86 |
| **Other lipids** | | Phosphatidylglycerol |  | -0.017 | 0.013 | 0.20 |  | 0.023 | 0.057 | 0.68 |
|  |  | Phosphatidylcholine |  | -0.016 | 0.013 | 0.23 |  | 0.022 | 0.057 | 0.70 |
|  |  | Total cholines |  | -0.019 | 0.013 | 0.15 |  | 0.014 | 0.055 | 0.81 |
|  |  | Sphingomyelin |  | -0.023 | 0.012 | 0.061 |  | -0.0013 | 0.053 | 0.98 |
| **Fatty adids** | Absolute | Total fatty acids |  | -0.017 | 0.014 | 0.21 |  | 0.037 | 0.058 | 0.53 |
|  |  | Unsaturation% |  | -0.010 | 0.013 | 0.42 |  | -0.0040 | 0.055 | 0.94 |
|  |  | SAFA |  | -0.014 | 0.014 | 0.31 |  | 0.045 | 0.059 | 0.45 |
|  |  | MUFA |  | -0.0072 | 0.013 | 0.58 |  | 0.012 | 0.056 | 0.83 |
|  |  | PUFA |  | -0.030 | 0.014 | **0.028** |  | 0.036 | 0.058 | 0.54 |
|  |  | Omega-3 |  | -0.023 | 0.013 | 0.077 |  | 0.058 | 0.055 | 0.29 |
|  |  | DHA |  | -0.020 | 0.013 | 0.11 |  | 0.035 | 0.054 | 0.52 |
|  |  | Omega-6 |  | -0.028 | 0.014 | **0.040** |  | 0.026 | 0.058 | 0.65 |
|  |  | LA |  | -0.025 | 0.014 | 0.070 |  | 0.019 | 0.059 | 0.75 |
|  | Proportions | SAFA% |  | 0.0038 | 0.014 | 0.78 |  | 0.050 | 0.058 | 0.39 |
|  |  | MUFA% |  | 0.0086 | 0.012 | 0.47 |  | -0.020 | 0.050 | 0.70 |
|  |  | PUFA% |  | -0.0043 | 0.012 | 0.73 |  | -0.0065 | 0.053 | 0.90 |
|  |  | Omega-3% |  | -0.020 | 0.013 | 0.12 |  | 0.064 | 0.055 | 0.24 |
|  |  | DHA% |  | -0.018 | 0.012 | 0.14 |  | 0.026 | 0.053 | 0.63 |
|  |  | Omega-6% |  | -0.0011 | 0.012 | 0.93 |  | -0.027 | 0.053 | 0.62 |
|  |  | LA% |  | -0.0053 | 0.012 | 0.66 |  | -0.032 | 0.052 | 0.55 |
| **Glycolysis** | | Glucose |  | -0.017 | 0.013 | 0.21 |  | -0.055 | 0.057 | 0.34 |
|  |  | Lactate |  | -0.0072 | 0.013 | 0.58 |  | 0.11 | 0.055 | **0.042** |
|  |  | Citrate |  | 0.00068 | 0.013 | 0.96 |  | -0.11 | 0.056 | **0.044** |
|  |  | Glycerol |  | 0.0088 | 0.016 | 0.57 |  | 0.047 | 0.066 | 0.47 |
| **Ketone bodies** | | Pyruvate |  | -0.0088 | 0.013 | 0.50 |  | 0.13 | 0.055 | **0.018** |
|  |  | Beta-hydroxybutyrate |  | 0.0019 | 0.014 | 0.89 |  | -0.026 | 0.061 | 0.67 |
|  |  | Acetate |  | 0.019 | 0.014 | 0.18 |  | 0.039 | 0.061 | 0.52 |
|  |  | Acetoacetate |  | 0.0081 | 0.014 | 0.57 |  | -0.073 | 0.061 | 0.23 |
| **Fluid balance** | | Albumin |  | -0.011 | 0.014 | 0.44 |  | 0.026 | 0.061 | 0.68 |
|  |  | Creatinine |  | -0.011 | 0.012 | 0.37 |  | -0.082 | 0.052 | 0.11 |
| **Inflammation** | | Glycoprotein acetyls |  | -0.0083 | 0.013 | 0.53 |  | 0.079 | 0.056 | 0.15 |
| **Amino acids** |  | Alanine |  | -0.023 | 0.013 | 0.070 |  | 0.053 | 0.055 | 0.34 |
|  |  | Glutamine |  | 0.0047 | 0.014 | 0.73 |  | 0.059 | 0.059 | 0.32 |
|  |  | Glysine |  | -0.0084 | 0.012 | 0.47 |  | -0.013 | 0.049 | 0.79 |
|  |  | Histidine |  | -0.016 | 0.014 | 0.28 |  | -0.017 | 0.061 | 0.79 |
|  |  | Phenylalanine |  | -0.0077 | 0.014 | 0.57 |  | 0.051 | 0.058 | 0.38 |
|  |  | Tyrosine |  | -0.0092 | 0.013 | 0.49 |  | 0.044 | 0.057 | 0.44 |
|  | Branched chain | Isoleucine |  | -0.0075 | 0.013 | 0.56 |  | 0.022 | 0.055 | 0.69 |
|  |  | Leucine |  | -0.016 | 0.013 | 0.21 |  | 0.0056 | 0.055 | 0.92 |
|  |  | Valine |  | -0.0093 | 0.013 | 0.48 |  | 0.027 | 0.056 | 0.63 |
